# A deep eutectic solvent mediated synthesis of spiro[naphthalene-2,5′-pyrimidine]-4-carbonitriles and their *in silico* inhibitory potential against the SARS-CoV-2 main protease

**DOI:** 10.1039/d6ra02921c

**Published:** 2026-07-03

**Authors:** Ankita Chaudhary, Pooja Saluja, Divya Mathur, Sushma Yadav

**Affiliations:** a Department of Chemistry, Maitreyi College, University of Delhi Delhi-110021 India achaudhary@maitreyi.du.ac.in; b Department of Chemistry, Daulat Ram College, University of Delhi Delhi-110007 India; c Department of Chemistry, Hansraj College, University of Delhi Delhi-110007 India

## Abstract

A green and efficient deep eutectic solvent (DES)-mediated multicomponent strategy has been developed for the synthesis of spiro[naphthalene-2,5′-pyrimidine]-4-carbonitriles. This protocol utilizes a biodegradable choline chloride/urea (1 : 2) DES as a sustainable reaction medium, and offers a good yield of targeted products (67–93%), broad substrate scope and high atom economy, while eliminating volatile organic solvents and harsh conditions. The DES can be easily recovered and reused for multiple cycles with minimal loss of efficiency. With operational simplicity, straightforward isolation and excellent recyclability, this approach offers practical and environmental benign multicomponent synthesis of spiro[naphthalene-2,5′-pyrimidine]-4-carbonitriles. The synthesized compounds were also docked and molecular dynamics (MD) simulations was performed to investigate the binding affinity, molecular interactions and stability of our synthesized spiro[naphthalene-2,5′-pyrimidine]-4-carbonitriles with the amino acids of coronavirus main protease (6LU7). All the compounds were found to be potent in comparison with reference inhibitor, remdesivir (−6.62 kcal mol^−1^) with binding energies ranging from −7.53 to −9.98 kcal mol^−1^. Overall, the present study demonstrates an efficient and sustainable DES-mediated strategy for the synthesis of biologically important spiro[naphthalene-2,5′-pyrimidine]-4-carbonitrile derivatives with promising antiviral potential against SARS-CoV-2 Mpro. The integration of green synthetic methodology with favourable *in silico* biological evaluation highlights the medicinal significance of this approach and its potential to promote future antiviral drug discovery research.

## Abstract 2

DES-mediated synthesis of spiro[naphthalene-2,5′-pyrimidine]-4-carbonitriles exhibiting potent SARS-CoV-2 main protease inhibitory potential.

## Graphical abstract

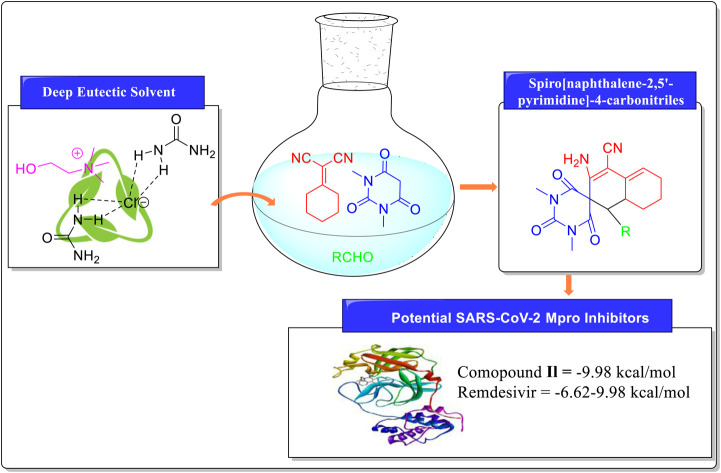

## 1. Introduction

The global impact of the COVID-19 pandemic has catalysed an urgent search for effective antiviral agents, specifically targeting the functional proteins of the Severe Acute Respiratory Syndrome Coronavirus 2 (SARS-CoV-2).^1^ Among these targets, the main protease (M^pro^), also known as chymotrypsin-like cysteine protease (3CLpro), plays a critical role in the viral life cycle by mediating the replication and transcription of the virus. Because M^pro^ lacks a human homolog, it represents an ideal target for drug design, offering a high degree of selectivity and a reduced risk of side effects in patients.^2^

Parallel to the need for therapeutic innovation is the growing demand for green chemistry in the pharmaceutical industry. The principles of green chemistry have stemmed from a conceptual paradigm into a pivotal framework in modern synthetic organic chemistry, primarily motivated by the urgent need to mitigate the environmental impact of industrial chemical methodologies. Green chemistry aims to design sustainable, energy-efficient and environmentally benign methodologies that decrease or eliminate the use and generation of hazardous substances, thereby minimizing environmental impact. The evolution of Multicomponent Reactions (MCRs) stands as a cornerstone of this transition, and offers a synthetic strategy that allows the construction of highly functionalized molecules starting from three or more materials in a single reaction step. MCRs offer a streamlined approach that maximizes atom economy as well as reduces energy consumption.^3,4^ The architecture of modern drug discovery is built upon the foundation of heterocyclic chemistry, where the ability to rapidly synthesize diverse molecular libraries is paramount.^5,6^

Among various heterocyclic synthons, barbituric acid (2,4,6-trioxypyrimidine) is widely recognised as a privileged scaffold due to its noticeable chemical reactivity and its ability to generate various biologically relevant compounds.^7–9^ First synthesised by Adolf von Baeyer in 1864, barbituric acid contains a pyrimidine core bearing three carbonyl groups. The high acidity of barbituric acid's C-5 methylene protons (p*K*_a_ ≈ 4.01), facilitates facile deprotonation, tautomerism and nucleophilic reactivity and makes it an ideal synthon for various MCRs. Though barbituric acid itself demonstrates limited biological activity, functionalisation at C-5 position has resulted in a wide range of biologically relevant barbiturates. The introduction of Barbital (Veronal) in 1903, the first hypnotic agent of its class, marked the beginning of barbiturate-based therapeutics, followed by Phenobarbital (Luminal), which remains a gold-standard anticonvulsant for the treatment of epilepsy globally. Structural modification of the barbiturate framework governs pharmacological behaviour; for example, phenyl substitution enhances anticonvulsant activity, while oxygen-to-sulfur replacement at the C-2 position affords Thiopental, an ultra-short-acting anaesthetic.^10^ In addition, barbituric acid derivatives continue to find utility in combination therapies, exemplified by Butalbital in analgesic formulations.^11^

From a synthetic perspective, barbituric acid readily participates in Knoevenagel condensations, Michael additions and cascade cyclisation processes, which serve as the foundation for the synthesis of structurally intricate systems such as pyrano[2,3-*d*]pyrimidines, pyrido[2,3-*d*]pyrimidines, furo[2,3-*d*]pyrimidines and spiro-barbiturates. In particular, spiro-barbiturates-characterized by a rigid spiro junction linking the barbiturate core to an additional cyclic framework have garnered increasing attention in chemical community, due to their distinct three dimensionality, enhanced conformational rigidity and promising pharmacological application including antibacterial,^12^ antifungal,^12^ anti-convulsant, hypnotics, anaesthetics,^13^ and anti-cancer agents.^14^ The synthetic routes that are available for the construction of spiro[naphthalene-2,5′-pyrimidine]-4-carbonitriles involves the use of 1,8-diazabicyclo(5.4.0)undec-7-ene (DABCO), ethylene glycol, octadecyl-1,4-diazabicyclo[2.2.2]octane bromide ([C_18_-DABCO][Br]) under ultrasonication, 1-[3-(dimethylamino)propyl] 1,4-diazabicyclo[2.2.2]octan-1-ium hydroxide, 1-butyl-3-methylimidazolium hydroxide (Bmim[OH]), which have their own merits and drawbacks.^15–19^ DBU and DABCO suffer from recyclability issues, ethylene glycol is less sustainable due to its non-renewable origin and limited biodegradability, while ionic liquids are associated with high cost along with sustainability concerns. Moreover, systems employing [C_18_-DABCO][Br] require specialized ultrasound activation, which reduces operational simplicity. These drawbacks highlight the need for greener and more efficient alternatives. In this regard, deep eutectic solvents (DESs) have gained considerable attention as sustainable reaction media for the synthesis of heterocyclic scaffolds, often affording good to excellent yields under mild reaction conditions with simplified work-up procedures.^20–22^ DESs are typically formed by combination of a hydrogen bond acceptor, such as choline chloride, with a hydrogen bond donor, like urea, glycerol, or organic acids. The resulting hydrogen-bonding network leads to a significant depression in melting point thereby producing non-volatile, non-flammable, biodegradable solvents that are well suited for green synthetic protocols.^23^ These features highlight their potential as sustainable media in modern heterocyclic and medicinal chemistry.

In continuation of our efforts aimed at developing greener and more efficient synthetic protocols, the present work reports a deep eutectic solvent-mediated multicomponent strategy for the synthesis of novel spiro[naphthalene-2,5′-pyrimidine]-4-carbonitriles. Subsequently, molecular docking and molecular dynamics (MD) simulation studies were performed as a preliminary computational screening approach to evaluate possible interactions of the synthesized compounds with the SARS-CoV-2 main protease (Mpro).

## 2. Results and discussion

### 2.1. Chemistry

**Scheme 1:**
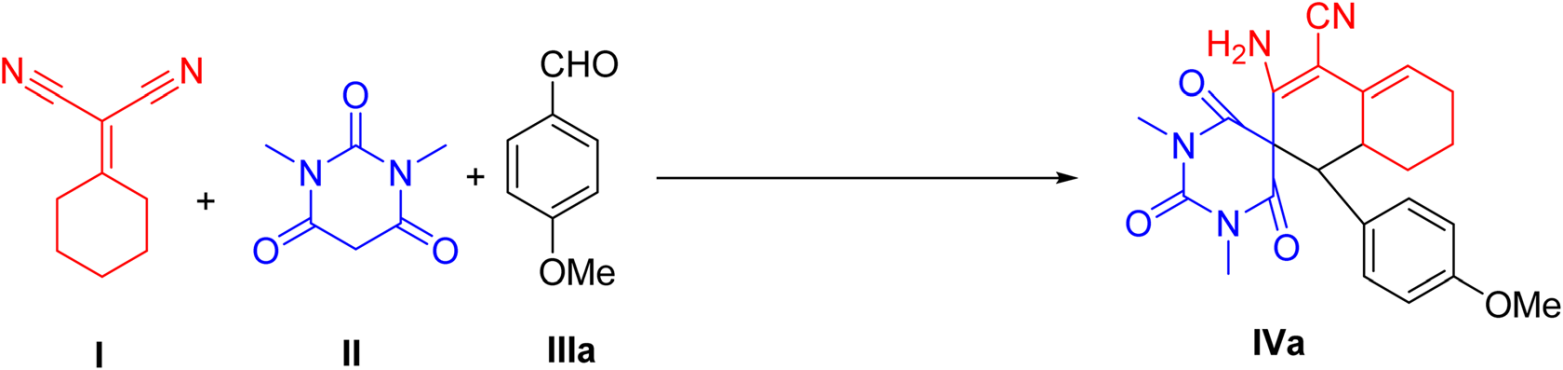
Synthesis of 3-amino-1-(4-methoxyphenyl)-1′,3′-dimethyl-2′,4′,6′-trioxo2′,3′,4′,6,6′,7,8,8a-octahydro-1*H*,1′*H*-spiro[naphthalene-2,5′-pyrimidine]-4-carbonitrile (IVa).

In this paper we have reported a green and sustainable strategy for the synthesis of spiro[naphthalene-2,5′-pyrimidine]-4-carbonitriles IVa–p by the reaction of electron-deficient α,α-dicyanoalkenes *i.e.* cyclohexylidene malononitrile (I), 1,3-dimethyl barbituric acid (II), and aromatic aldehydes (IIIa–p) in choline chloride/urea (1 : 2) DES at 80 °C. Initially, the model reaction of 1,3-dimethylbarbituric acid (1.0 mmol), 4-methoxybenzaldehyde (1.0 mmol) and cyclohexanone (1.0 mmol) and malononitrile (1.0 mmol) was attempted in ChCl/urea (1 : 2) at 80 °C. Under these conditions, the reaction remained incomplete even after 5 h and afforded 3-amino-1-(4-methoxyphenyl)-1′,3′-dimethyl-2′,4′,6′-trioxo2′,3′,4′,6,6′,7,8,8a-octahydro-1*H*,1′*H*-spiro[naphthalene-2,5′-pyrimidine]-4-carbonitrile (IVa) in 30% yield after separation by column chromatography (Table 1, entry 1). Next, the reaction was executed using preformed cyclohexylidenemalononitrile (1.0 mmol) (synthesized by reaction of cyclohexanone and malononitrile by reported procedure^24^) along with 1,3-dimethyl barbituric acid 1.0 mmol) and 4-methoxybenzaldehyde (1.0 mmol) in ChCl/urea (1 : 2) at 80 °C (Table 1, entry 2) (Scheme 1). The reaction proceeded smoothly to completion in 3 h and gave an excellent 93% of IVa (Table 1, entry 2). However, lowering the reaction temperature to 60 °C resulted in incomplete conversion even after 6 h, affording only 70% yield of IVa after workup, while increasing the temperature to 90 °C showed no significant improvement in either yield or reaction time (Table 1, entries 3–4). Further, to identify a suitable reaction medium, the optimised model reaction was evaluated using different deep eutectic solvents, namely ChCl/thiourea (1 : 2), ChCl/glucose (1 : 1), ChCl/malonic acid (1 : 1), ChCl/oxalic acid (1 : 1) (Table 1, entries 5–8), at 80 °C and furnished IVa in comparatively lower yield of 75%, 60%, 40% and 45%, respectively. Furthermore, the reactions performed in individual components of DES, *viz.* choline chloride and urea, were incomplete even after 8 h and showed multiple spots on TLC (Table 1, entries 9–10), clearly reinforcing the concept of DES-induced synergistic catalysis. Therefore, the optimal reaction conditions for the one-pot three-component condensation of cyclohexylidenemalononitrile with 1,3-dimethylbarbituric acid and 4-methoxybenzaldehyde involved the use of ChCl/urea (1 : 2) at 80 °C.

**Table 1:** Optimisation studies for the synthesis of 3-amino-1-(4-methoxyphenyl)-1′,3′-dimethyl-2′,4′,6′-trioxo 2′,3′,4′,6,6′,7,8,8a-octahydro-1*H*,1′*H*-spiro[naphthalene-2,5′-pyrimidine]-4-carbonitrile (IVa)

Entry	Reaction conditions	Temperature (°C)	Time (h)	Yield (%)^d^
1	ChCl/urea (1 : 2)	80	5	30^a^^,^^b^
2	ChCl/urea (1 : 2)	80	3	93^c^
3	ChCl/urea (1 : 2)	60	6	70^b^^,^^c^
4	ChCl/urea (1 : 2)	90	3	92^b^^,^^c^
5	ChCl/thiourea (1 : 2)	80	5	75^b^^,^^c^
6	ChCl/glucose (1 : 1)	80	5	60^b^^,^^c^
7	ChCl/malonic acid (1 : 1)	80	5	40^b^^,^^c^
8	ChCl/oxalic acid (1 : 1)	80	5	45^b^^,^^c^
9	ChCl	80	8	30^b^^,^^c^
10	Urea	80	8	25^b^^,^^c^

a Reaction of 1,3-dimethylbarbituric acid (1.0 mmol), 4-methoxybenzladehyde (1.0 mmol), and cyclohexanone (1.0 mmol), malononitrile (1.0 mmol) was carried out using 2.0 mL DES.

b Incomplete reaction.

c Reaction of cyclohexylidenemalononitrile (1.0 mmol), 1,3-dimethylbarbituric acid (1.0 mmol) and 4-methoxybenzaldehyde (1.0 mmol) was performed using 2.0 mL of DES.

d Isolated Yield.

**Scheme 2:**
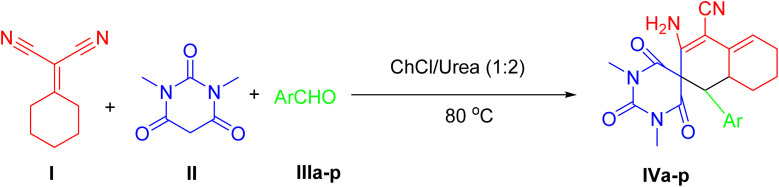
ChCl/urea (1 : 2) assisted synthesis of spiro[naphthalene-2,5′-pyrimidine]-4-carbonitriles (IVa–p).

Thereafter, on the basis of the above optimized reaction conditions, we extended our substrate scope towards evaluating the generality and applicability of this method using diversely substituted aldehydes. It was observed that aromatic aldehydes bearing both electron-withdrawing and electron-donating substituents readily underwent the reaction, affording the corresponding spiro[naphthalene-2,5′-pyrimidine]-4-carbonitriles (IVa–p) in good to excellent yields (Scheme 2). The results are summarized in Table 2. Notably, a relatively lower yield of the desired spiro product was obtained with thiophene-2-carboxaldehyde. This observation may be attributed to the comparatively lower conversion of the corresponding thiophene-derived arylidene barbituric acid intermediate into the final spiro product. Furthermore, reactions involving aliphatic aldehydes did not proceed to completion and resulted in complex product mixtures, which may be attributed to their lower electrophilic character compared to aromatic aldehydes.

**Table 2:** Synthesis of spiro[naphthalene-2,5′-pyrimidine]-4-carbonitriles (IVa–p) *via* reaction of 1,3-dimethylbarbituric acid (1.0 mmol), aldehydes (1.0 mmol) and cyclohexylidene malononitrile (1.0 mmol) in 2.0 mL ChCl/urea (1 : 2) at 80 °C

S. no.	Product	Ar	Yield (%)	Time (h)
1	IVa	4-OMeC_6_H_4_	93	3.0
2	IVb	4-MeC_6_H_4_	93	3.0
3	IVc	4-ClC_6_H_4_	86	2.5
4	IVd	4-FC_6_H_4_	85	2.5
5	IVe	3-ClC_6_H_4_	84	2.5
6	IVf	4-BrC_6_H_4_	92	2.5
7	IVg	4-NO_2_C_6_H_4_	80	2.5
8	IVh	3-BrC_6_H_4_	89	2.5
9	IVi	2-ClC_6_H_4_	90	3.0
10	IVj	3,4,5-(MeO)_3_C_6_H_2_	87	2.0
11	IVk	3-MeOC_6_H_4_	88	2.5
12	IVl	4-(Me)_2_C_6_H_4_	93	3.0
13	IVm	C_6_H_5_	87	3.5
14	IVn	4-CNC_6_H_4_	90	2.5
15	IVo	2-Thienyl	67	3.5
16	IVp	4-CF_3_C_6_H_4_	86	2.5

**Scheme 3:**
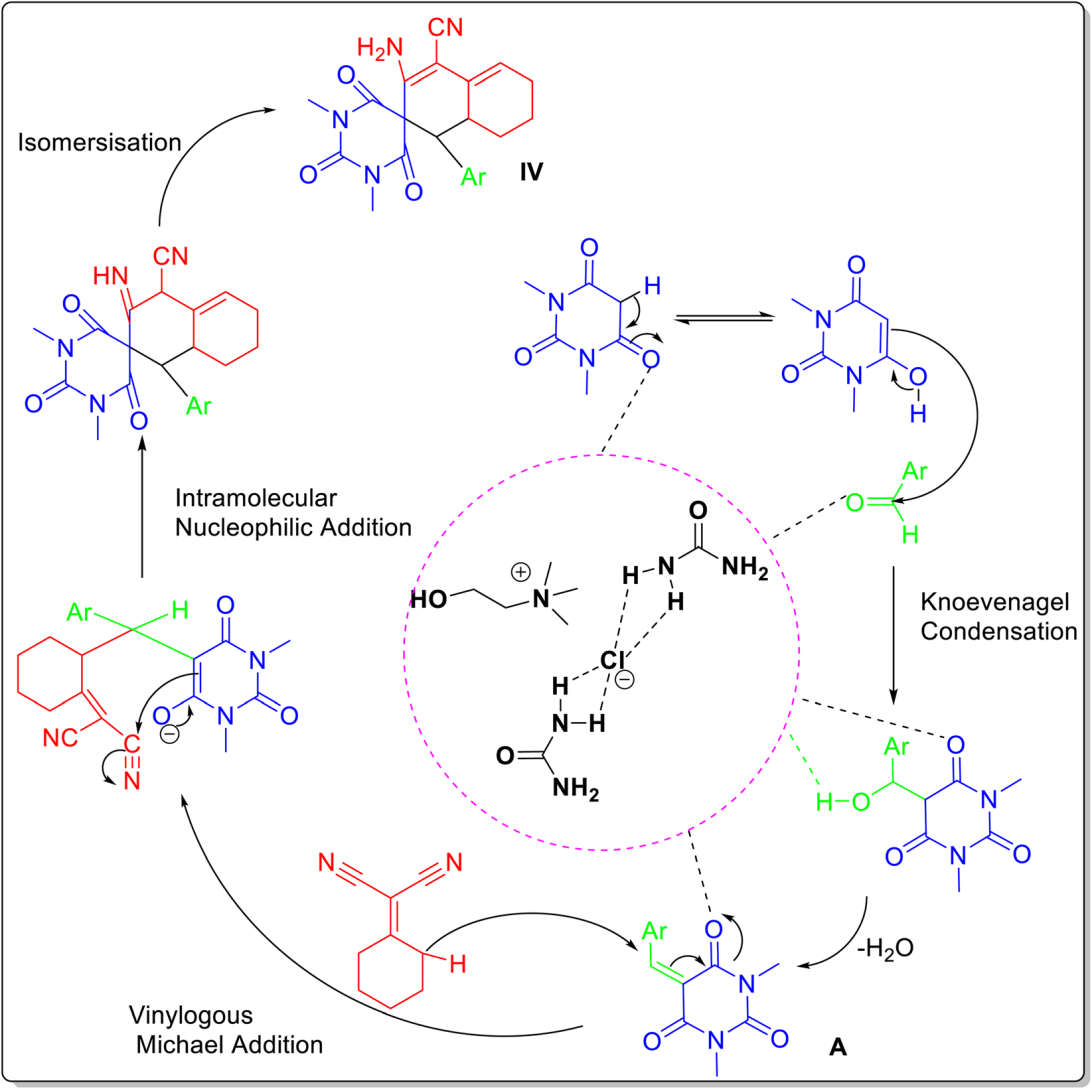
Proposed mechanism for ChCl/Urea (1 : 2) assisted synthesis of spiro[naphthalene-2,5′-pyrimidine]-4-carbonitriles (IV).

The formation of spiro[naphthalene-2,5′-pyrimidine]-4-carbonitriles (IV) can be rationalized *via* a domino reaction pathway as depicted in Scheme 3. Overall, ChCl/Urea deep eutectic solvent functions as a green, bifunctional reaction medium, simultaneously activating electrophile, stabilizing the charged intermediates and avoiding the need for an external catalyst. ChCl/Urea activates aldehyde and 1,3-dimethylbarbituric acid, promoting Knoevenagel condensation to form the Michael acceptor A. Simultaneously, the DES assists in γ-deprotonation of cyclohexylidenemalononitrile, generating a stabilized carbanion that undergoes Michael addition to A, affording an intermediate which thereby undergoes intramolecular addition followed by isomerization to furnish spiro[naphthalene-2,5′-pyrimidine]-4-carbonitrile IV.

Compared to the previously reported protocols, ChCl/urea (1 : 2) system afforded the highest product (IVf) yield (92%) under relatively mild conditions with shorter reaction time, while avoiding toxic solvents, specialized catalysts and techniques, thereby demonstrating superior efficiency and sustainability (Table 3).

**Table 3:** Comparison of the reported methods for the synthesis of spiro[naphthalene-2,5′-pyrimidine]-4 carbonitriles

S. no.	Reaction conditions	Product, IVf yield (%)	Time (h)	Ref.
1	DBU, EtOH, rt^a^	90	3	18
2	Ethylene glycol, 100 °C^a^	82	2.5	19
3	[C_18_-DABCO][Br]), H_2_O, ultrasound^a^	84	2	20
4	1-[3-(Dimethylamino)propyl][′//1,4-diazabicyclo[2.2.2]octan-1-ium hydroxide, EtOH : H_2_O (1 : 1), r.t^b^	88	3	17
5	Bmim[OH], 80 °C^a^	85	1	21
6	Choline chloride/urea (1 : 2), 80 °C^a^	92	2.5	Present work

a Reaction conditions: 1,3-dimethylbarbituric acid (1.0 mmol), 4-bromobenzaldehyde (1.0 mmol), cyclohexylidene malononitrile (1.0 mmol).

b Reaction conditions: 1,3-dimethylbarbituric acid (1.0 mmol), 4-bromobenzaldehyde (1.0 mmol), cyclohexanone (1.0 mmol), malononitrile (1 mmol).

**Fig. 1:**
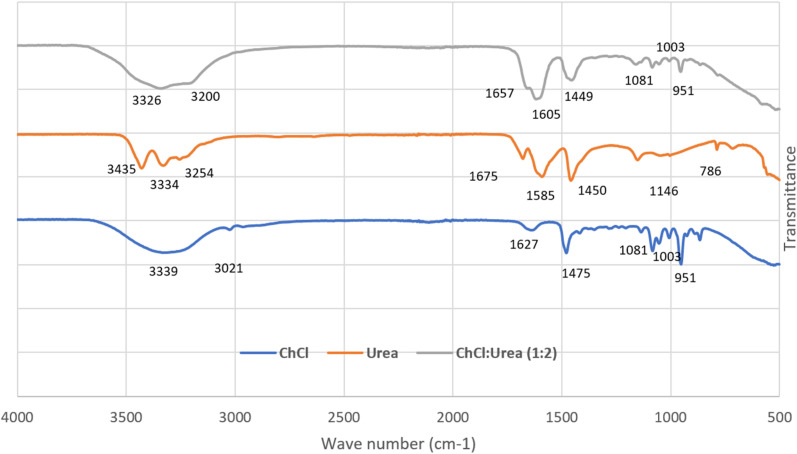
Comparative FTIR spectra of ChCl, urea and ChCl/urea(1 : 2).

FTIR spectra of the synthesised DES (ChCl/urea (1 : 2)) and its individual components of DES *viz.* choline chloride and urea are shown in Fig. 1. The persistence of characteristic ChCl peaks including *ρ* CH_3_ (1481 cm^−1^), *ρ* CH_2_ (1081 cm^−1^), *ν* C–O (1003 cm^−1^), *ν*_as_ CCO (951 cm^−1^) in ChCl/urea DES indicates that the structure of Ch^+^ was retained without structural degradation in ChCl/urea DES. The urea N–H stretching bands at 3435 cm^−1^, 3334 cm^−1^ and 3254 cm^−1^, corresponding to N–H stretching vibrations of urea, broaden notably in the DES, indicative of an extensive hydrogen-bonding network between urea and choline chloride. In addition, the N–H and C=O stretching vibrations of urea in DES showed a slight shift to a lower wavenumber, signifying hydrogen bond formation between urea's –NH_2_ and Cl of ChCl.

The recyclability of choline chloride/urea (1 : 2) was also evaluated using the model reaction of cyclohexylidenemalononitrile (1.0 mmol), 1,3-dimethylbarbituric acid (1.0 mmol) and 4-bromobenzaldehyde (1.0 mmol) at 80 °C, over three consecutive cycles without adding fresh DES. After each run, the product was precipitated by addition of water and the solid was isolated by filtration and the DES was recovered by evaporation of water under reduced pressure. The ChCl/urea (1 : 2) DES demonstrated excellent recyclability, delivering yields of 92%, 90%, and 88% with corresponding reaction times of 2.5, 2.5, 3 h for the first, second, and third cycles, respectively, indicating minimal loss of catalytic efficiency upon reuse.

Additionally, to demonstrate the scalability and practical applicability of the developed DES-mediated approach, reaction of cyclohexylidenemalononitrile (10.0 mmol), 1,3-dimethylbarbituric acid (10.0 mmol) and 4-bromobenzaldehyde (10.0 mmol) at 80 °C in 20 mL DES, we observed no significant drop in yield (91% *vs.* 92%) and no increase in reaction time, suggesting the method is amenable to scale-up.

### 2.2. *In silico* investigations of potential inhibitor of SARS-CoV-2 M^pro^ using molecular docking and molecular dynamic simulation

#### 2.2.1 Molecular docking study

The main protease plays a vital role in the protease-mediated virus maturation, as it cleaves viral polyproteins into functional proteins required for replication. The inhibition of M^pro^ blocks this process and effectively suppresses viral replication. The molecular docking analysis provides the details of binding mode, essential amino acid residues of the binding site, and the intermolecular interactions of the experimentally synthesized drug candidates with the binding site of 3CLpro main protease. In addition, MD simulation investigates the stability of binding interactions obtained through docking analyses.

**Fig. 2:**
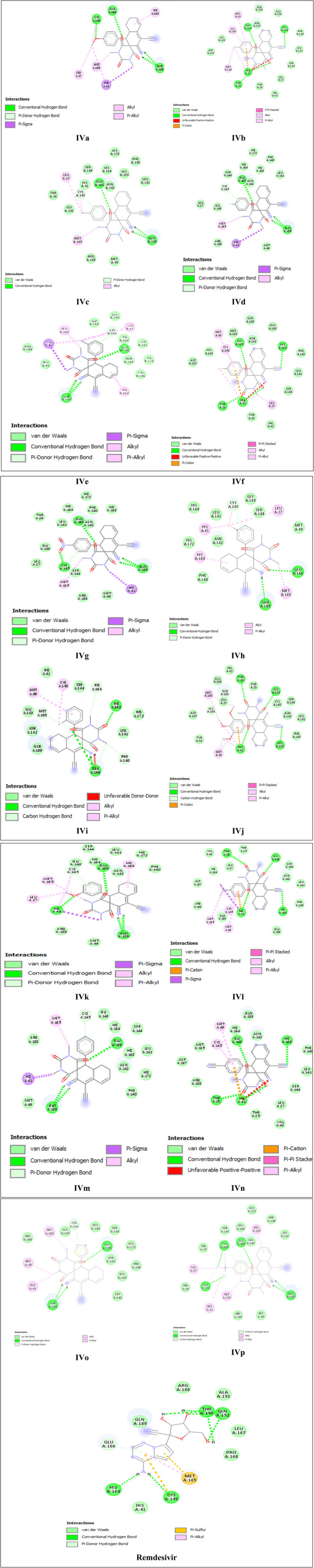
2D model interactions between 3CLpro and synthesized compounds IVa–p and remdesivir. The nature of protein–ligand interactions is shown with different color legends.

Molecular docking simulations were performed for the synthesized spiro[naphthalene-2,5′-pyrimidine]-4-carbonitrile ligands against the SARS CoV-2 main protease (M^pro^/3CLpro (PDB ID: 6LU7). Table 4 summarizes the binding affinities (Δ*G* in kcal mol^−1^) of the complex obtained by the best docked structure of each ligand with 3CLpro. As shown in Table 4, the hit compounds docking scores against the 3CLpro ranging from −7.53 to −9.98 kcal mol^−1^ (Fig. 2). Notably, compound IVl exhibited a more favorable predicted binding energy toward the SARS-CoV-2 main protease (Mpro) compared with Remdesivir (−6.62 kcal mol^−1^) using the employed AutoDock simulation conditions. Remdesivir, used here as a reference ligand, has been clinically investigated for the treatment of SARS-CoV-2 infections.^25^ However, the obtained docking energies represent only computational estimations of protein–ligand interactions and do not directly establish inhibitory potency or therapeutic superiority. Furthermore, remdesivir primarily exerts its antiviral activity through inhibition of the viral RNA-dependent RNA polymerase (RdRp), while the present study evaluates the potential interactions of the synthesized compounds with SARS-CoV-2 Mpro. Accordingly, the comparison with remdesivir is intended only as a computational reference for relative docking behavior rather than as evidence of an identical antiviral mechanism. Therefore, the docking results should be interpreted only as preliminary indicators of possible binding affinity and require further biochemical and cellular validation. The docking analysis provides preliminary structural insight into possible ligand-Mpro interactions and may assist future structural optimization of spiro-based derivatives targeting SARS-CoV2-Mpro for antiviral screening.

**Table 4:** Thermodynamic binding affinities of spiro[naphthalene-2,5′-pyrimidine]-4-carbonitrile derivatives (IV) against SARS-CoV-2 3CLpro main protease receptor (PDB ID: 6LU7). Δ*G* corresponds to free energy of binding of the complex obtained by the best docked pose of each ligand structure with protein

Compound	Chemical structure	Δ*G* (kcal mol^−1^)
IVa	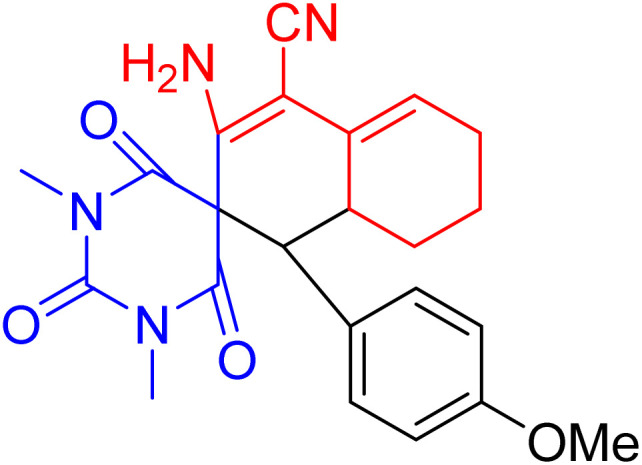	−9.27
IVb	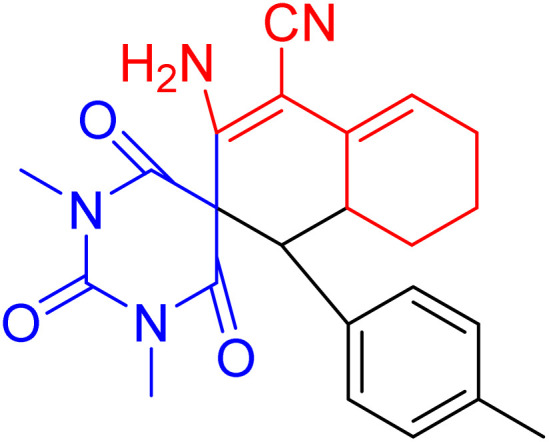	−8.92
IVc	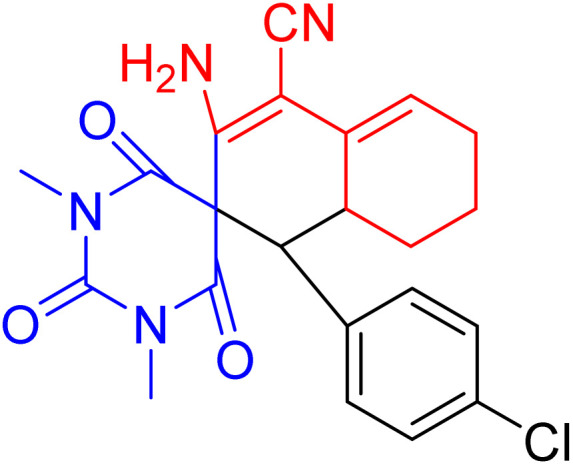	−9.00
IVd	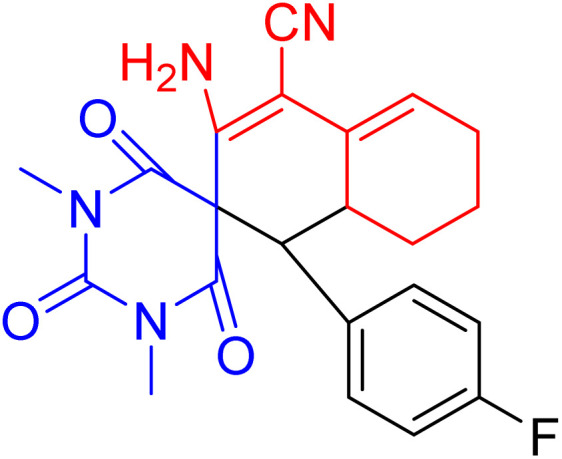	−8.39
IVe	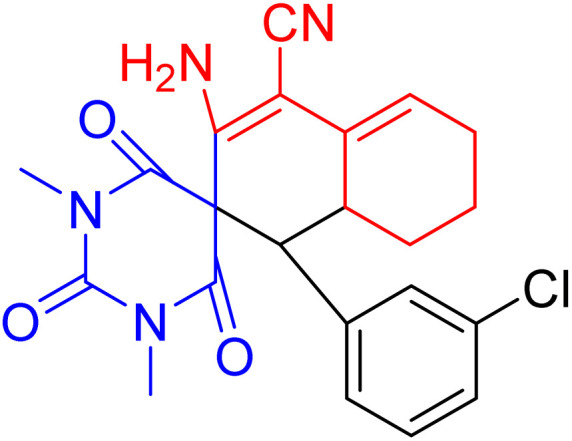	−8.84
IVf	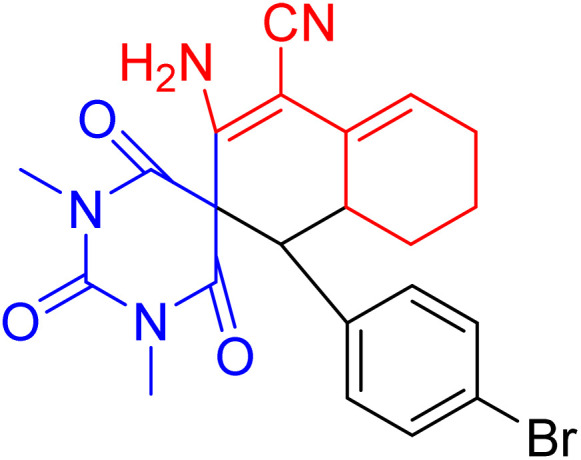	−9.37
IVg	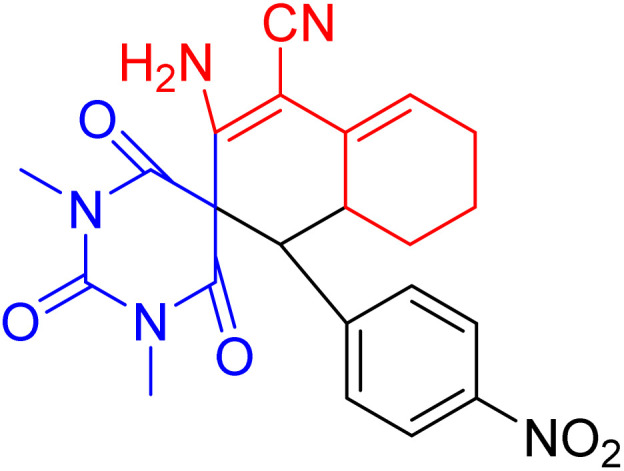	−9.30
IVh	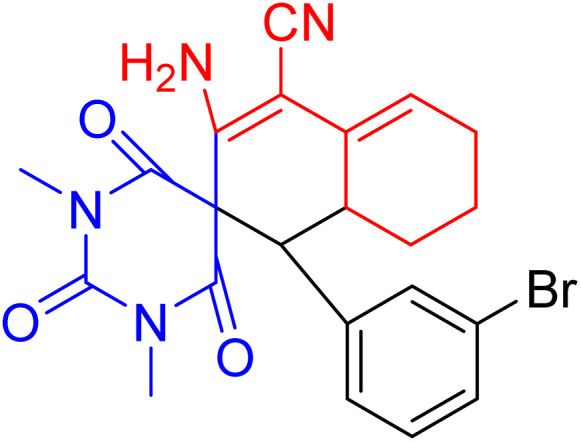	−8.83
IVi	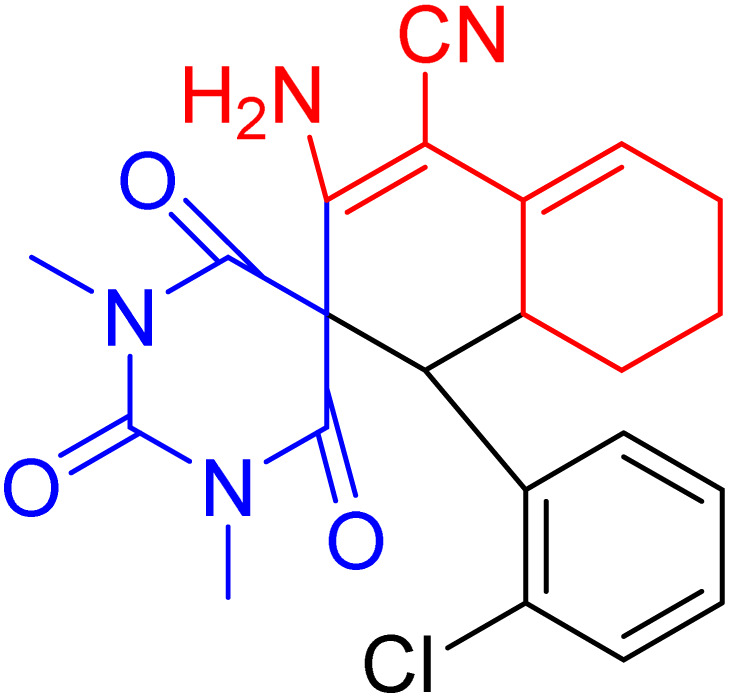	−7.53
IVj	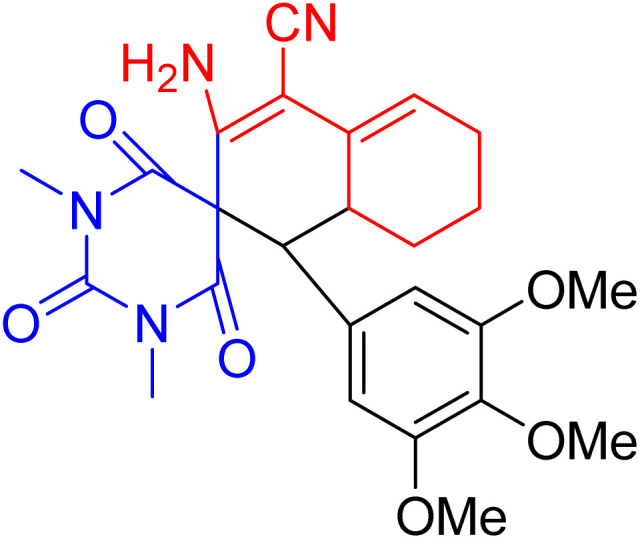	−8.25
IVk	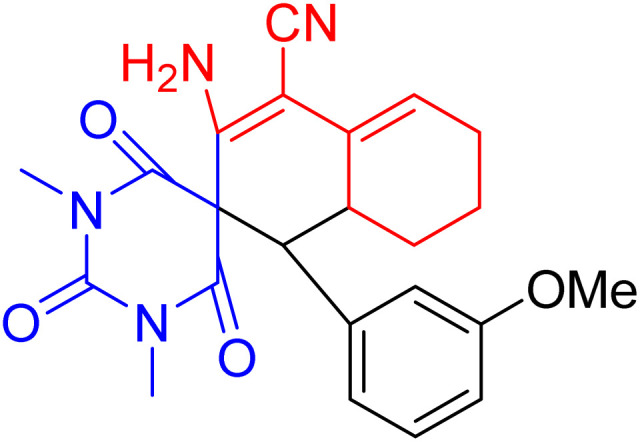	−8.68
IVl	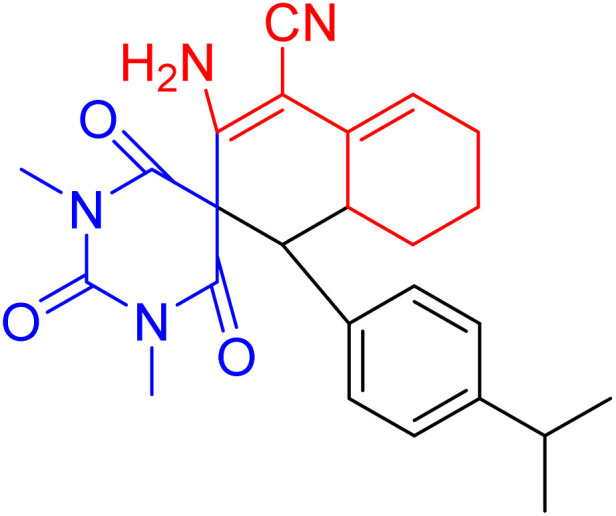	−9.98
IVm	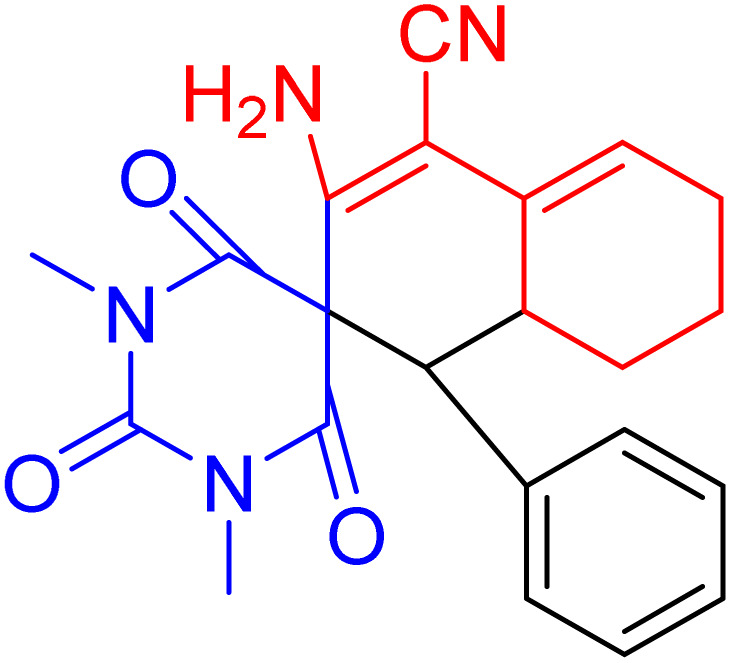	−8.32
IVn	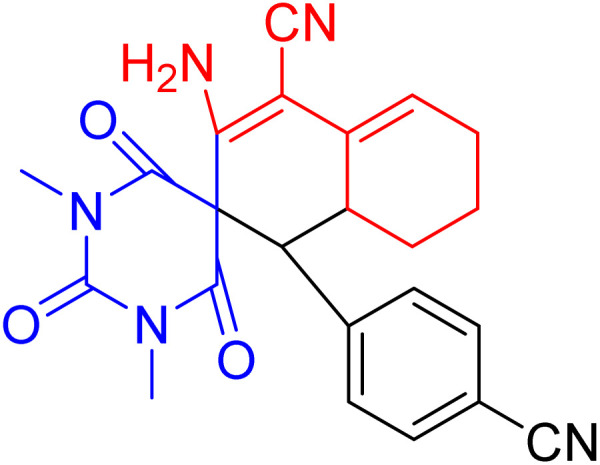	−9.27
IVo	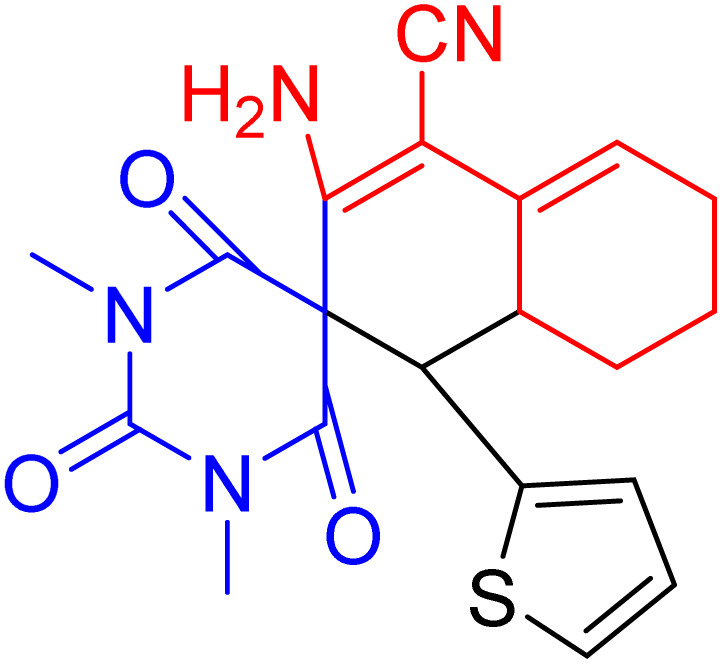	−8.23
IVp	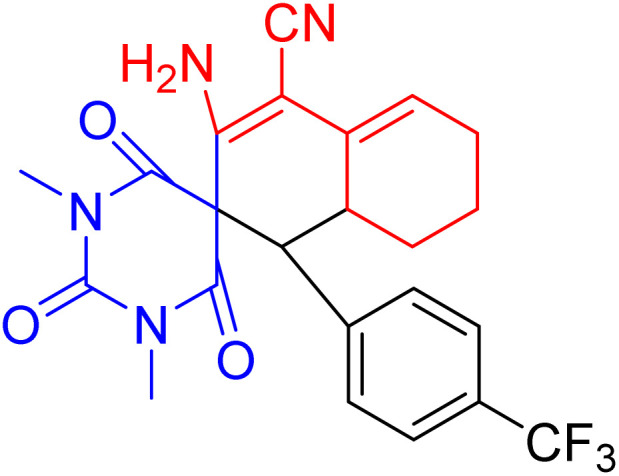	−8.68
Remdesivir	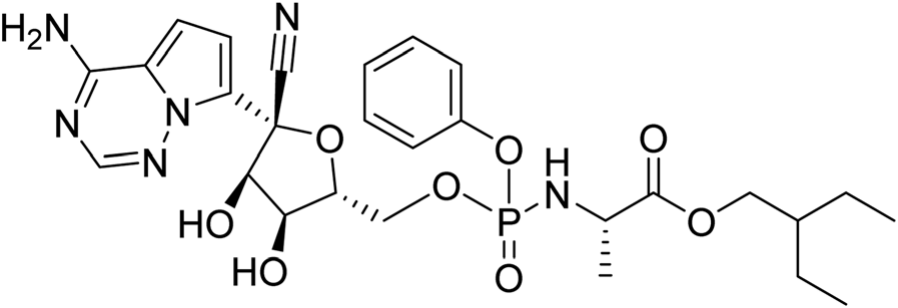	−6.62

**Fig. 3:**
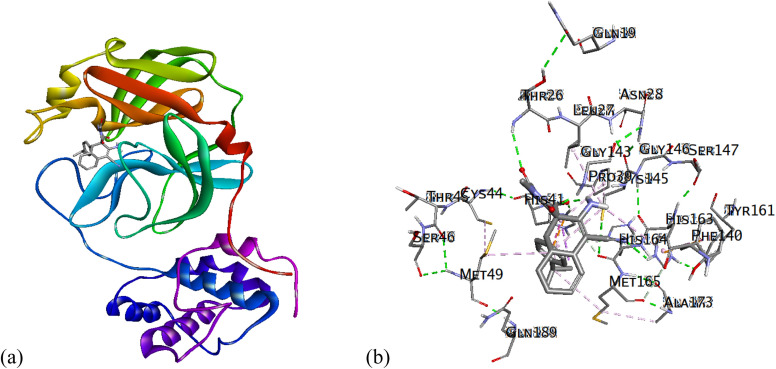
(a) 3-Dimensional model of the best hit compound, 4l and 3CLpro (b) binding interactions between IVl and 3CLpro visualized using Biovia Discovery studio Visualizer.

Among the screened derivatives, the compound exhibiting the most favorable binding affinity (−9.98 kcal mol^−1^) was selected for detailed interaction analysis. As illustrated in Fig. 3(a), IVl was perfectly sequestered within a well-defined catalytic cavity of the 3CLpro enzyme. The stability of the protein–ligand complex is reinforced by a network of specific interactions.

Four conventional hydrogen bonds were identified. Three of these occur between the carbonyl groups (C=O) of the ligand and residues HIS41, THR26 and GLY143. A fourth hydrogen bond is formed between the positively charged HIS44 and the nitrile group of IVl. In addition, histidine (HIS41) forms one π–cation bond with the benzyl group and one π–sigma bond. Two π–alkyl bonds were observed between the benzyl ring and the hydrophobic residues, methionine (MET49) and cysteine (CYS145). Additionally, an alkyl interaction was noted between methionine (MET165) and the ligand's alkyl group. These interactions align with previously recognized drug-binding pockets, confirming the potential of this scaffold as a potent 3CLpro inhibitor.^26–28^ The results obtained from these combined experimental and theoretical studies provide key ideas to future drug design approaches to develop potential compounds that inhibit the SARS-CoV-2 3CLpro main protease activity.

#### 2.2.2 Molecular dynamics simulation

**Fig. 4:**
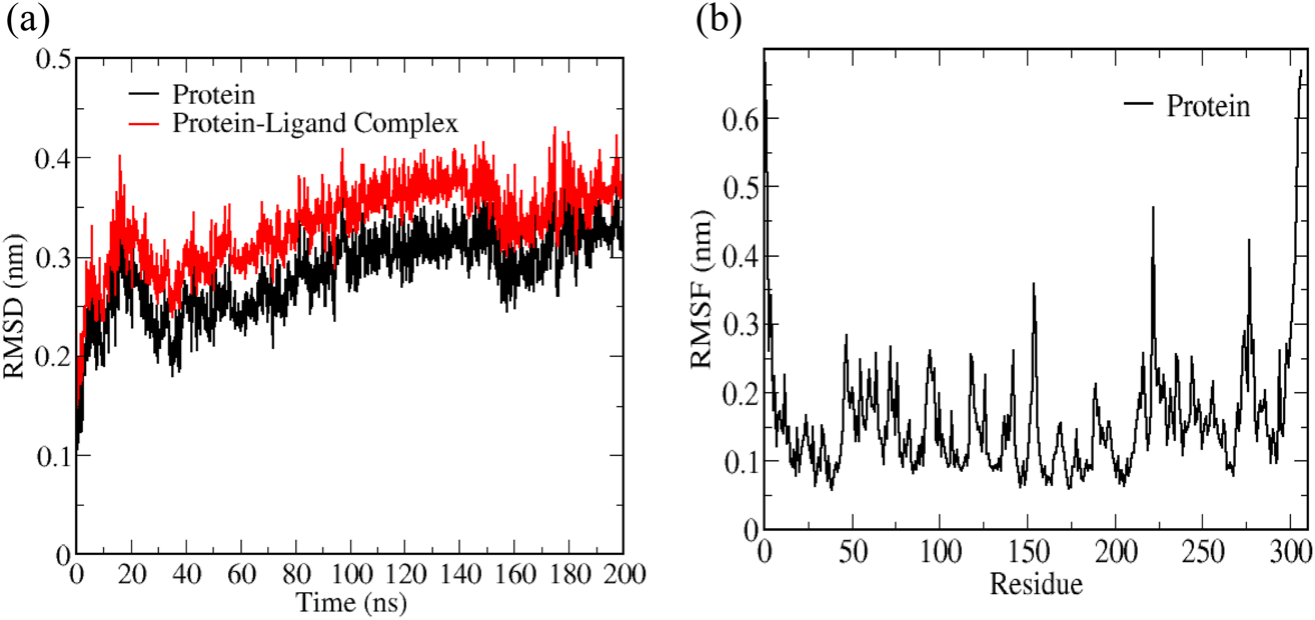
(a) Root Mean Square Deviation (RMSD) of 3CLpro-IVl complex; (b) Root Mean Square Fluctuation (RMSF) of 3CLpro residues are less than 0.6 nm.

To validate the conformational stability and binding consistency of IVl with the SARS CoV-2 3CLpro enzyme, a 200 ns molecular dynamics simulation was performed. Root mean square deviation (RMSD) was utilized to measure the deviation of the protein backbone from its initial structural conformation. Generally, smaller deviations signify a more stable protein–ligand system. As shown in Fig. 4a, both systems reached a state of equilibrium after the initial 40 ns.

The RMSD of the 3CLpro-ligand complex equilibrated at approximately 4 Å and remained nearly constant throughout the remainder of the 200 ns trajectory. While the compound exhibited slightly higher RMSD values compared to the protein, indicating localized structural flexibility, the overall consistency confirms a stable ligand binding profile with 3CLpro protein.

To assess stability at the residue level, root mean square fluctuation (RMSF) was analyzed. The residues maintained stable fluctuations with values remaining below 0.6 nm (Fig. 4b).

The stability of 3CLpro-4l complex is further supported by literature, which defines the active site residues HIS41, CYS145, MET49, MET165, GLY143, THR26, and GLU166 amino acids as critical anchors for 3CLpro inhibition.^26–28^ Consequently, these results suggest that this structure offers a promising framework for identifying novel drug candidates against coronavirus *via* virtual screening tools such as Autodock.

## 3. Experimental

### 3.1. Chemistry

Commercially available analytical-grade chemicals and solvents from Merck and Sigma-Aldrich were used as received without further purification. Reaction progress was monitored using Silica gel 60 F254 Pre-coated Aluminium plates from Merck. Melting points were determined on Tropical Labequip apparatus and are uncorrected. IR (KBr) spectra were recorded on a PerkinElmer FTIR spectrophotometer, and the values are expressed as *ν*_max_/cm^−1^. The ^1^H and ^13^C spectra were recorded on Jeol JNM ECX-400P at 400 MHz and 100 MHz, respectively. Chemical shift values are recorded on *δ* scale, and the coupling constants (*J*) are in Hertz. The alkylidine malononitrile was prepared by reported procedure.^24^ The DES *viz*. ChCl/urea, ChCl/thiourea, ChCl/malonic acid, ChCl/oxalic acid, ChCl/glucose, were prepared by heating the two components in the required molar ratio under continuous, vigorous stirring until a clear, homogeneous liquid was obtained; the resulting DESs were used without further purification.^29–33^

#### 3.1.1 General procedure for the synthesis of spiro[naphthalene-2,5′-pyrimidine]-4-carbonitriles (IVa–p)

A mixture of 1,3-dimethylbarbituric acid (1.0 mmol), aromatic aldehyde (1.0 mmol) and cyclohexylidenemalononitrile (1.0 mmol), was taken in a 50 mL round-bottomed flask containing DES (2.0 mL). The reaction contents were stirred magnetically in an oil bath maintained at 80 °C for the appropriate time as indicated in Table 2. The progress of the reaction was monitored by TLC using ethyl acetate: petroleum ether, (30 : 70, v/v) as eluent. Upon completion of the reaction (Table 2), the mixture was quenched with water (∼5 mL). The resulting precipitate was collected by vacuum filtration, washed thoroughly with water and recrystallized with ethanol to afford the pure products (IVa–p).

### 3.2. *In silico* simulations analysis

The 3D crystal structure of the main protease in complex with the inhibitor N3 (PDB ID: 6LU7) (https://www.rcsb.org/)^34^ of SARS-CoV-2 was retrieved from Protein Data Bank. The protein was prepared for the docking process by removing water molecules and the co-crystal inhibitor and then protonated by adding polar hydrogen using MGLTOOLS 1.5.6 (https://mgltools.scripps.edu/). Charges to the protein and drug compounds were assigned using the Kollman united atom and Gasteiger charge method, respectively.

Molecular docking was carried out using AutoDock 4.2 program through Autodock Tools 1.5.6 (ADT).^35^ A grid box with a size of *x* = 66, *y* = 74, and *z* = 74 and centered at *x* = −12.849, *y* = 12.987, *z* = 68.818 was defined to cover the N3 binding pocket (substrate binding site) in the 3CLpro enzyme with 0.342 Å grid points spacing assigned with default atomic solvation parameters. Lamarckian Genetic Algorithm was selected as a docking engine, with the running times of genetic algorithms set at 100, and the maximum number of evaluations and the maximum number of generations were set to 2 500 000 and 27 000, respectively. All other parameters were set at the default value. The conformer with the lowest binding energy was considered as the best binding mode for the most stable protein–ligand complex. The molecular interactions and binding modes of the top poses were visually examined for interaction using Discovery Studio Visualizer, v.24.1.0.23298, (BIOVIA, Dassault Systèmes, 2024).

#### 3.2.1 Molecular dynamics simulation

Molecular dynamics simulation was conducted to determine dynamics of the docked protein–ligand complex with the best docking score and get affirmation on the drug binding mode stability in the specified simulation time. The protein was parameterized using the AMBER ff14SB force field, while topology for the ligand was generated using the General Amber Force Field (GAFF) with AM1-BCC partial charges assigned *via* ACPYPE. The molecular dynamics simulation of the complex was performed using the GROMACS (version 2020.1) simulation module.^36^ The complex was solvated using the TIP3P water model in a cubic box by keeping the complex in the center with 1 nm distance from the box edge. The required number of Na^+^ and Cl^−^ ions added to make the simulation system electrically neutral. Long-range electrostatics were treated using the Particle Mesh Ewald (PME) method with a real-space cutoff of 10 Å. A non-bonded interaction cutoff of 10 Å was also applied. Bond lengths involving hydrogen atoms were constrained using the SHAKE algorithm, allowing an integration time step of 2 fs. The energy minimization was carried out using steepest descent minimization algorithm followed by two step equilibrations of NVT (constant number of particles, volume and temperature) and NPT (constant number of particles, pressure, and temperature) for 1 ns each. The temperature was maintained at 300 K using the Nose–Hoover thermostat with a coupling constant of 1 ps, while pressure was maintained at 1 bar using the Parrinello–Rahman barostat with a coupling constant of 2 ps. The production run under NVT thermostat was then performed at 300 K for a duration of 200 ns. Trajectory analysis was performed using GROMACS built-in tools to compute root-mean-square deviation (RMSD) and root-mean-square fluctuation (RMSF) to analyze the complex stability over the simulation time. The Xmgrace program was used to make the plots and visual molecular dynamics (VMD) used for visualizations to represent the analyses.^37^

## 4. Conclusion

In conclusion, an efficient and environmentally benign deep eutectic solvent–mediated multicomponent strategy has been developed for the synthesis of spiro[naphthalene-2,5′-pyrimidine]-4-carbonitriles. The use of choline chloride/urea (1 : 2) provides a green reaction medium that enables high yields under mild conditions thereby obviating the need of volatile organic solvents. The protocol offers broad substrate scope, excellent atom economy and easy product isolation. Moreover, the DES could be readily recovered and reused over multiple cycles without a significant decrease loss in efficiency, highlighting the sustainability and practical applicability of the method. Overall, this approach epitomizes a valuable contribution to green multicomponent synthesis for the construction of complex heterocyclic frameworks. The antiviral potential of the synthesized spiro[naphthalene-2,5′-pyrimidine]-4-carbonitriles were evaluated against the SARS-CoV-2 M^pro^ using a combination of molecular docking and molecular dynamics simulations. The results indicate that these compounds bind effectively to the M^pro^ target. Consequently, this study provides a strategic framework for designing and synthesizing new libraries of barbituric acid-based-compounds, which may lead to the development of highly effective treatment for COVID-19. Further experimental validation through IC_50_ measurements, protease inhibition assays, and antiviral cell-based studies will be necessary to confirm the predicted interactions.

## Author contributions

A. C. conceived and supervised the project. A. C. and P. S. carried out the synthesis and experimental work. S. Y. performed the *in silico* studies. The manuscript was written by A. C., D. M., and S. Y. All authors reviewed and approved the final manuscript.

## Conflicts of interest

The authors declare no conflicts of interest, financial or otherwise.

## Supplementary Material

RA-OLF-D6RA02921C-s001

## Data Availability

The data supporting the findings of this study are available within the article and its supplementary information (SI). Supplementary information: complete characterisation data of all the synthesised compounds including IR, ^1^H NMR, ^13^C NMR and HRMS data. See DOI: https://doi.org/10.1039/d6ra02921c.
