# Supplementary material for: A deep eutectic solvent mediated synthesis of spiro[naphthalene-2,5′-pyrimidine]-4-carbonitriles and their in silico inhibitory potential against the SARS-CoV-2 main protease
Source: RSC Adv. 2026 Jul 3;16(35):35222–34. doi: 10.1039/d6ra02921c (PMC13329696; doi:10.1039/d6ra02921c)

Supplementary Information (SI) for RSC Advances.
This journal is © The Royal Society of Chemistry 2026

A Deep Eutectic Solvent Mediated Synthesis of Spiro[naphthalene-2,5′-pyrimidine]-4-carbonitriles and their *In Silico* Inhibitory Potential against the SARS-CoV-2 Main Protease

Ankita Chaudhary,*[a] Pooja Saluja,*[a] Divya Mathur,[b] Sushma Yadav [c]

[a] *Department of Chemistry, Maitreyi College, University of Delhi, Delhi-110021*

[a] *Department of Chemistry, Daulat Ram College, University of Delhi, Delhi-110007*

[a] *Department of Chemistry, Hansraj College, University of Delhi, Delhi-110007*

Email: achaudhary@maitreyi.du.ac.in

Spectral Data of spiro[naphthalene-2,5'-pyrimidine]-4-carbonitriles (IVa-p)

3-Amino-1-(4-methoxyphenyl)-1',3'-dimethyl-2',4',6'-trioxo-2',3',4',6,6',7,8,8a-octahydro-1*H*,1'*H*-spiro[naphthalene-2,5'-pyrimidine]-4-carbonitrile, IVa ($C_{23}H_{24}N_4O_4$)

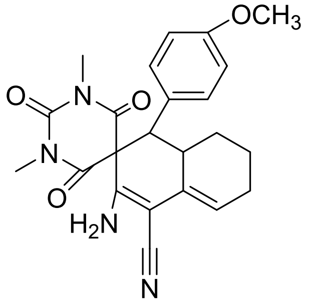

White solid; M.p.: 254-256°C (Lit.: 253-255°C); IR (ν_{max}, cm$^{-1)}$ (DCM): 3348, 2201, 1665, 1426, 1380; ^1H NMR (400 MHz, DMSO-d_6) δ = 6.87-6.77 (m, 2H, ArH) 6.75-6.71 (m, 2H, ArH), 6.45 (s, 2H, NH_2), 5.55 (s, 1H, vinyl), 3.66 (s, 3H, OCH_3), 3.35 (m, 1H, merged with solvent), 2.97-2.94 (m, 1H), 2.88 (s, 3H, CH_3), 2.86 (s, 3H, CH_3), 2.19-1.96 (m, 2H), 1.63-1.60 (m, 1H), 1.33-1.31 (m, 2H), 0.73-0.61 (m, 1H); ^{13}C NMR (DMSO-d_6) δ = 168.7, 167.2, 159.1, 151.4, 149.6, 131.2, 129.9, 126.8, 126.6, 117.6, 117.2, 114.4, 113.3, 82.3, 61.2, 55.6, 55.2, 32.3, 28.1, 27.6, 25.0, 21.5. HRMS (ESI) calcd. for $C_{23}H_{24}N_4O_4$ $[M+H]^+$: 421.1876, found: 421.1865 $[M+H]^+$.

3-Amino-1',3'-dimethyl-2',4',6'-trioxo-1-*p*-tolyl-2',3',4',6,6',7,8,8a-octahydro-1*H*,1'*H*spiro[naphthalene-2,5'-pyrimidine]-4-carbonitrile, IVb ($C_{23}H_{24}N_4O_3$)

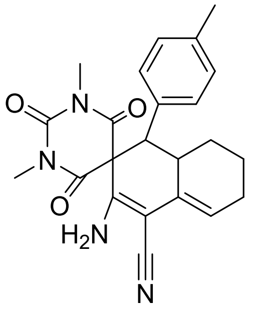

White solid; M.p.: 240-242°C (Lit.: 241-243°C); IR (ν_{max}, $cm^{-1)}$ (DCM): 3334, 3231, 2205, 1654, 1426, 1370; ^{1}H NMR (400 MHz, DMSO-d_6) δ = 7.15 (d, 1H, *J*= 8.0 Hz, Ar-H), 7.07 (d, 1H, *J*=8.0 Hz, Ar-H), 6.81 (d, 1H, *J*= 8.0 Hz, Ar-H), 6.73 (d, 1H, *J*=8.0 Hz, Ar-H), 6.49 (s, 2H, -NH_2), 5.5 (s, 1H, vinylic), 3.06-3.02 (m, 1H, -CH), 2.91-2.81 (m, 7H, 2(CH_3) + CH), 2.23(s, 3H, CH_3), 2.18-2.08 (m, 2H), 1.64 (bs, 1H, -CH_2), 1.37-1.34 (m, 2H, -CH_2), 0.77-0.68 (m, 1H, -CH_2); ^{13}C NMR (DMSO-d_6) δ = 169.2, 167.5, 151.5, 150.2, 138.1, 132.1, 131.3, 130.6, 129.3, 126.0, 118.2, 118.0, 82.8, 61.3, 56.3, 32.3, 28.6, 28.5, 27.9, 25.3, 21.2, 21.1. HRMS (ESI) calcd. for $C_{23}H_{24}N_4O_3$ $[M+H]^+$: 405.1848, found: 405.1924 $[M+H]^+$.

3-Amino-1-(4-chlorophenyl)-1',3'-dimethyl-2',4',6'-trioxo-1',3',4',6,6',7,8,8a-octahydro-1H,2'H-spiro[naphthalene-2,5'-pyrimidine]-4-carbonitrile, IVc ($C_{22}H_{21}ClN_4O_3$)

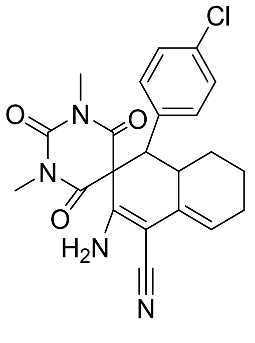

White solid; M.p.: 284-286°C (Lit.: 286-288°C); IR (ν_{max}, $cm^{-1)}$ (DCM): 3322, 3205, 2200, 1678, 1428, 1378; ^{1}H NMR (400 MHz, DMSO-d_6) δ = 7.42 (d, 1H, *J*= 8.0 Hz, Ar-H), 7.35 (d, 1H, *J*= 8.0 Hz, Ar-H), 6.96 (d, 1H, *J*= 8.00 Hz, Ar-H), 6.91 (d, 1H, *J*= 8.0 Hz, Ar-H), 6.52 (s, 2H, -NH_2), 5.62 (s, 1H, vinylic), 3.34 (m, 1H, merged with solvent), 3.11-3.08 (m, 1H, -CH), 2.94-2.91 (m, 6H, 2(CH_3)), 2.18-2.06 (m, 2H), 1.65 (bs, 1H, - CH_2), 1.38-1.31 (m, 2H, -CH_2), 0.78-0.75 (m, 1H, -CH_2); ^{13}C NMR (DMSO-d_6) δ = 168.9, 167.3, 151.3, 150.1, 134.4, 133.4, 132.6, 131.1, 128.8, 128.2, 117.8, 117.4, 82.11, 60.6, 55.1, 32.2, 28.7, 28.6, 27.8, 25.3, 21.9.

HRMS (ESI) calcd. for $C_{22}H_{21}ClN_4O_3$ $[M + H]^+$: 425.1375, found: 425.1378 $[M + H]^+$, 427.1352 $[M + H + 2]^+$.

3-Amino-1-(4-fluorophenyl)-1',3'-dimethyl-2',4',6'-trioxo-2',3',4',6,6',7,8,8a-octahydro-1*H*,1'*H*-spiro[naphthalene-2,5'-pyrimidine]-4-carbonitrile, IVd ($C_{22}H_{21}FN_4O_3$)

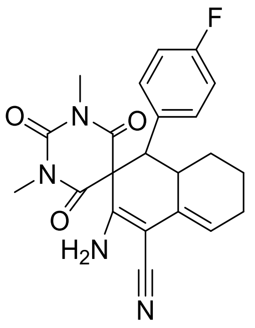

White solid; M.p.: 254-256°C (Lit.: 258-260°C); IR (v_{max}, $cm^{-1)}$ (DCM): 3331, 3301, 2202, 1681, 1426, 1376; ^{1}H NMR (400 MHz, DMSO-d_6) δ = 7.17-7.05 (m, 2H, ArH), 6.94-6.87 (m, 2H, ArH), 6.48 (s, 2H, NH_2), 5.56 (s, 1H, vinyl), 3.35 (m, 1H, merged with solvent), 3.05 (d, 1H, J = 12.4 Hz), 2.88 (s, 3H, $-CH_3$), 2.87 (s, 3H, $-CH_3$), 2.13-2.02 (m, 2H), 1.60 (bs, 1H), 1.37-1.27 (m, 2H), 0.77-0.67 (m, 1H); ^{13}C NMR (DMSO-d_6) δ = 168.4, 167.1, 161., 151, 149.8, 132.4, 131, 130.8, 128.2, 128, 117.8, 117.4, 115.3, 82.2, 60.9, 55, 32.4, 28.2, 27.3, 24.9, 21.6 HRMS (ESI) calcd. for $C_{22}H_{21}FN_4O_3$ $[M + H]^+$: 409.1676, found: 409.1682 $[M+H]^+$.

3-Amino-1-(3-chlorophenyl)-1',3'-dimethyl-2',4',6'-trioxo-1',3',4',6,6',7,8,8a-octahydro-1*H*,2'*H*-spiro[naphthalene-2,5'-pyrimidine]-4-carbonitrile, IVe ($C_{22}H_{21}ClN_4O_3$)

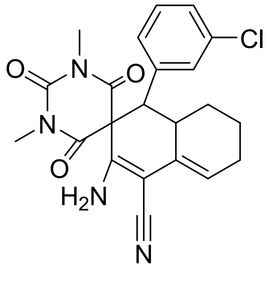

White solid; M.p.: 274-276°C (Lit.: 275-277 °C);); IR (v_{max}, $cm^{-1)}$ (DCM): 2192, 1672, 1443, 1379; ^{1}H NMR (400 MHz, DMSO-d_6) δ = 7.36-7.26 (m, 2H, Ar-H), 6.93 (s, 1H, Ar-H), 6.91-6.85 (m, 1H, Ar-H), 6.53 (s, 2H, $-NH_2$), 5.61 (s, 1H, vinylic), 3.36-3.35 (m, 1H, -CH), 3.1 (d, 1H, J= 12.21 Hz, -CH), 2.17-2.13 (m, 1H, $-CH_2$), 2.04-2.03 (m, 1H, $-CH_2$), 1.66-1.65 (m, 1H,

-CH_2), 1.43- 1.33 (m, 2H, -CH_2), 0.84-0.73 (m, 1H, -CH_2); ^{13}C NMR (DMSO-d_6) δ = 168.5, 166.0, 151.1, 149.5, 137.6, 133.4, 133, 130.2, 129, 128.5, 128.1, 117.9, 117.6, 82, 60.5, 55.4, 32.2, 28.1, 28.2, 27.6, 25.1, 21.6. HRMS (ESI) calcd. for $C_{22}H_{21}ClN_4O_3$ $[M+ Na]^+$: 447.1194, found: 447.1191 $[M+Na]^+$, 448.1214$[M+Na+1]^+$.

3-Amino-1-(4-bromophenyl)-1',3'-dimethyl-2',4',6'-trioxo-1',3',4',6,6',7,8,8a-octahydro-1*H*,2'*H*-spiro[naphthalene-2,5'-pyrimidine]-4-carbonitrile, IVf ($C_{22}H_{21}BrN_4O_3$)

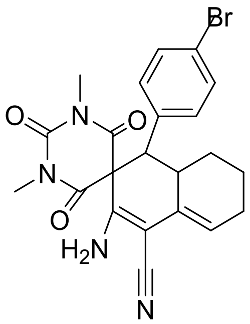

White solid; M.p.: 262-264°C (Lit.: 261-263°C); IR (ν_{max}, cm^{-1}) (DCM): 3326, 3209, 2198, 1672, 1419, 1371; ^{1}H NMR (400 MHz, DMSO-d_6) δ = 7.55 (d, 1H, *J*= 8 Hz, Ar-H), 7.48 (d, 1H, *J*= 8.0 Hz, ArH), 6.90 (d, 1H, *J*= 8.00 Hz, Ar-H), 6.84 (d, 1H, *J*= 8.0 Hz, Ar-H), 6.52 (s, 2H, -NH_2), 5.62 (s, 1H, vinylic), 3.36-3.34 (m, 1H, -CH), 3.09 (d, 1H, *J*= 12 Hz, -CH), 2.94-2.91 (m, 6H, 2(CH_3), 2.18-2.06 (m, 2H), 1.65 (bs, 1H), 1.38-1.30 (m, 2H, -CH_2), 0.78-0.75 (m, 1H, -CH_2); ^{13}C NMR (DMSO-d_6) δ = 168.9, 167.3, 151.2, 150.1, 134.7, 132.9, 131.8, 131.7, 130.9, 128.5, 121.9, 118.1, 117.5, 82.8, 61.1, 55.8, 32.59, 28.6, 27.8, 25.3, 21.9. HRMS (ESI) calcd. for $C_{22}H_{21}BrN_4O_3$ $[M + Na]^+$: 491.0689, found: 491.0687 $[M+Na]^+$, 493.0672 $[M+Na+2]^+$.

3-amino-1',3'-dimethyl-1-(4-nitrophenyl)-2',4',6'-trioxo-2',3',4',6,6',7,8,8a-octahydro1*H*,1'*H*-spiro[naphthalene-2,5'-pyrimidine]-4-carbonitrile, IVg ($C_{22}H_{21}N_5O_4$)

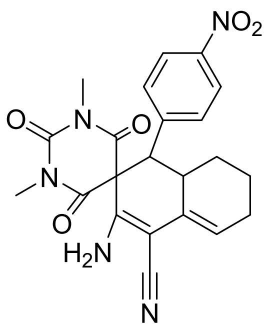

Pale Yellow solid; M.p.: 250-254°C(Lit.: 251-253); IR (ν_{max}, cm^{-1}) (KBr): 2208, 1683, 1431, 1352; ^{1}H NMR (400 MHz, DMSO-d$_6$) δ = 8.16- 8.11 (m, 2H, Ar-H), 7.20-7.16 (m, 2H, Ar-H), 6.53 (s, 2H, NH_2), 5.61 (s, 1H, vinyl), 3.24 (d, 1H, *J*= 12.3 Hz, -CH), 2.96-2.94 (m, 1H, -CH), 2.91 (s, 3H, -CH_3), 2.88 (s, 3H, -CH_3), 2.15-2.10 (m, 1H, -CH_2), 2.02-1.96 (m, 1H, -CH_2), 1.62-1.61 (m, 1H, -CH_2), 1.40-1.36 (m, 1H, -CH_2), 1.24-122 (m, 1H, -CH_2), 0.82-0.71 (m, 1H, -CH_2); ^{13}C NMR (DMSO-d$_6$) δ = 168.1, 166.8, 150.8, 149.6, 147.3, 143.1, 132.1, 130.3, 127.9, 123.6, 123.5, 117.8, 117.5, 82.4, 60.6, 55.3, 32.1, 28.4, 27.2, 24.9, 21.6; HRMS (ESI) calcd. for $C_{22}H_{21}N_5O_5$ [M + H]$^+$: 435.1561, found: 435.1556[M + H]$^+$.

3-Amino-1',3'-dimethyl-2',4',6'-trioxo-1-(3-bromophenyl)-2',3',4',6,6',7,8,8a-octahydro-1*H*,1'*H*spiro[naphthalene-2,5'-pyrimidine]-4-carbonitrile, Ivh ($C_{22}H_{21}BrN_4O_3$)

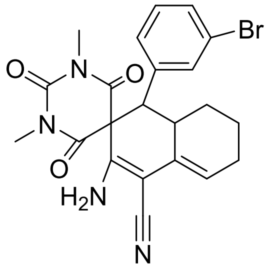

White solid; M.p. (°C): 251-253; IR ($CHCl_3$) ν_{max}/cm^{-1}: 2203, 1672, 1431, 1386, 742; ^{1}H NMR (400 MHz, DMSO-d$_6$) δ = 7.51-7.46 (m, 1H), 7.31-7.25 (m, 1H), 7.09-7.04 (m, 1H), 6.96-6.91 (m,1H), 6.53 (s, 2H, NH_2), 5.62 (s, 1H), 3.33 (m, 1H, merged with solvent), 3.09-2.98(m, 1H), 2.93-2.90 (m, 6H, (2CH_3)), 2.15-2.07 (m, 2H), 1.66 (bs, 1H), 1.45-1.28 (m, 2H), 0.87-0.73(m, 1H); ^{13}C NMR (DMSO-d$_6$) δ = 168.9, 168.8, 167.4, 167.2, 151.2, 150.05, 138.1, 133.2, 131.7, 130.8, 129.9, 129.1, 125.6, 121.9, 118.1, 82.6, 61.8, 55.3, 32.3, 28.2, 24.9, 21.5. HRMS (ESI) calcd. for $C_{22}H_{21}BrN_4O_3$ [M + H]$^+$: 468.0797, found: 469.0899 [M+H]$^+$, 471.0882 [M+H+2]$^+$.

Amino-1-(2-chlorophenyl)-1',3'-dimethyl-2',4',6'-trioxo-2',3',4',6,6',7,8,8a-octahydro-1H,1'H-spiro[naphthalene-2,5'-pyrimidine]-4-carbonitrile, Ivi ($C_{22}H_{21}ClN_4O_3$))

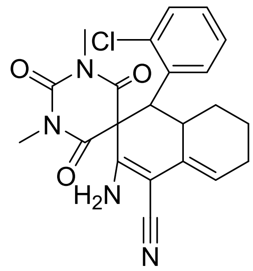

White solid; M.p.: 242-244°C (Lit.: 242-244°C); IR (ν_{max}, cm^{-1}) (DCM): 3364, 3248, 2206, 1752, 1680, 1594, 1432, 1376; ^{1}H NMR (400 MHz, DMSO-d_6) δ = 7.41 (d, 1H, J = 8.0 Hz, ArH), 7.33-7.24 (m, 2H, ArH), 6.99 (d, 1H, J = 8.0 Hz , 6.52 (s, 2H, NH_2), 5.59 (s, 1H, vinylic), 3.68 (d, 1H, J = 12Hz), 3.41-3.37 (m, 1H, merged with solvent), 2.90 (s, 3H, CH_3), 2.88 (s, 3H, CH_3), 2.16-1.97 (m, 2H), 1.60-1.49 (m, 1H), 1.35-1.16 (m, 2H), 0.72-0.60 (m, 1H); ^{13}C NMR (DMSO-d_6) δ = 168.7, 168.4, 167.5, 166.6, 151.1, 150.1, 137.7, 133.8, 131.0, 130.5, 129.5, 128.8, 126.2, 117.9, 82.5, 61.1, 55.9, 32.4, 28.5, 27.6, 25.3, 21.7. HRMS (ESI) calcd. for C_{22} $H_{21}ClN$ $_4O$ $_3$ $[M + Na]^+$: 447.1194, found: 447.1198 $[M+Na]^+$.

3-Amino-1',3'-dimethyl-2',4',6'-trioxo-1-(3,4,5-trimethoxyphenyl)-2',3',4',6,6',7,8,8a-octahydro-1*H*,*1'H*-spiro[naphthalene-2,5'-pyrimidine]-4-carbonitrile, Ivj ($C_{25}H_{28}N_4O_6$)

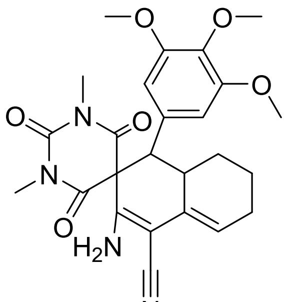

White solid; M.p.: 285-286°C (Lit.: 286-288°C); IR (ν_{max}, $cm^{-1)}$ (DCM): 3358, 3232, 2197, 1678, 1424, 1376; ^{1}H NMR (400 MHz, DMSO-d_6) δ = 6.49 (s, 2H, NH_2), 6.16 (s, 2H, ArH), 5.61 (s, 1H, vinyl), 3.70-3.68 (s, 6H, OCH_3), 3.60 (s, 3H, OCH_3), 3.32 (m, 1H, merged with solvent), 3.03-3.00 (m, 1H), 2.98-2.94 (m, 6H, 2(CH_3)), 2.23-2.09 (m, 2H), 1.66-1.63 (m, 1H), 1.50-1.47 (m, 2H), 0.84-0.75 (m, 1H); ^{13}C NMR (DMSO-d_6) δ = 169.2, 167.7, 153.8, 152.8

151.2, 149.6, 137.5, 131.1, 130.8, 117.8, 117.3, 108.3, 103.1, 82.2, 61.2, 60.5, 56.5, 56.3, 32.5, 31.1, 28.7, 27.5, 25.8, 21.9. HRMS (ESI) calcd. for $C_{25}H_{28}N_4O_6$ $[M + Na]^+$: 503.1901, found: 503.1914 $[M+H]^+$.

3-amino-1-(3-methoxyphenyl)-1',3'-dimethyl-2',4',6'-trioxo-2',3',4',6,6',7,8,8a-octahydro1*H*,1'*H*-spiro[naphthalene-2,5'-pyrimidine]-4-carbonitrile, IVk ($C_{23}H_{24}N_4O_4$)

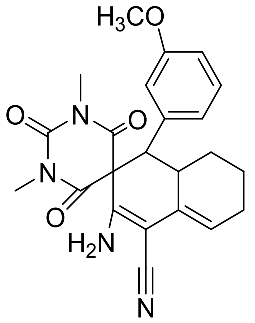

White solid; M.p. (°C): 242-244; IR ($CHCl_3$) ν_{max}/cm^{-1}: 2204, 1681, 1423, 1386; ^{1}H NMR (400 MHz, DMSO-d$_6$) δ = 7.27-7.16 (m, 1H, Ar-H), 6.99-6.83 (m, 1H, Ar-H), 6.49 (s, 2H, -NH_2), 6.44-6.42 (m, 2H, Ar-H), 5.61 (s, 1H, vinylic), 3.33-3.34 (m, 1H, merged with solvent), 3.71-3.68 (m, 3H, -OCH_3), 3.09-3.0 (m, 1H, -CH), 2.92-2.89 (m, 6H), 2.19-2.06 (m, 2H, -CH_2), 1.65 (bs, 1H), 1.44-1.38 (m, 2H, -CH_2), 0.72-0.84 (m, 1H, - CH_2); ^{13}C NMR (DMSO-d$_6$) δ = 169.1, 167.2, 159.8, 151.3, 150.2, 136.8, 131.2, 129.8, 122.8, 118.1, 116.3, 114.1, 111.8, 82.7, 61.3, 56.4, 55.4, 32.6, 28.5, 27.8, 25.3, 21.9. HRMS (ESI) calcd. for $C_{23}H_{24}N_4O_4$ $[M + Na]^+$: 443.1690, found: 443.1698 $[M+Na]^+$.

3-Amino-1-(4-isopropylphenyl)-1',3'-dimethyl-2',4',6'-trioxo-2',3',4',6,6',7,8,8a-octahydro-1H,1'H-spiro[naphthalene-2,5'-pyrimidine]-4-carbonitrile IVl ($C_{25}H_{28}N_4O_3$)

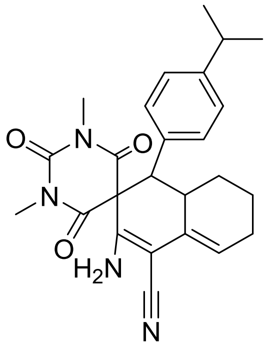

White solid; M.p.: 210-212°C (Lit.: 207-209°C); IR (ν_{max}, cm^{-1}) (DCM): 3352, 3251, 2208, 1673, 1426, 1372; ^{1}H NMR (400 MHz, DMSO-d$_6$) δ = 7.21 (d, 1H, J = 8.0 Hz), 7.11 (d, 1H, J

= 8.0 Hz) 6.85(d, 1H, J = 8.0 Hz), 6.76 (d, 1H, J = 8.0 Hz), 6.47 (s, 2H, NH_2), 5.60 (s, 1H, vinyl), 3.03-2.93 (m, 2H, CH_2), 2.87-2.86(m. 6H, 2(CH_3)), 2.83-2.78(m,1H, CH_2), 2.19-2.05 (m, 2H), 1.61 (bs, 1H), 1.44-1.41 (m, 2H), 1.14-1.13 (m, 6H, CH(CH_3)$_2$), 0.81-0.71 (m, 1H); ^{13}C NMR (DMSO-d$_6$) δ = 168.7, 167.1, 151.1, 149.5, 148.7, 132.0, 130.9, 130.1, 126.0, 125.9, 125.7, 117.8, 117.1, 82.1, 61.0, 55.8, 33.1, 32.0, 28.2, 28.1, 27.5, 24.9, 23.8, 23.7, 21.6 . HRMS (ESI) calcd. for $C_{25}H_{28}N_4O_3$ [M + Na]$^+$: 455.2053, found: 455.2064 [M+Na]$^+$.

3-Amino-1',3'-dimethyl-2',4',6'-trioxo-1-phenyl-2',3',4',6,6',7,8,8aoctahydro-1H,1'H-spiro[naphthalene-2,5'-pyrimidine]-4-carbonitrile, IVm ($C_{22}H_{22}N_4O_3$)

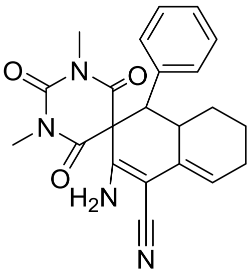

^{1}H NMR (400 MHz, DMSO-d$_6$) δ = 7.41-7.33 (m, 3H), 7.0-6.90 (m, 2H), 6.55 (s, 2H, NH_2), 5.67 (s, 1H, vinyl), 3.13-3.04 (m, 2H, CH_2), 3.01-2.94 (m. 6H, 2(CH_3)), 2.25-2.12 (m, 2H, CH_2), 1.71 (bs, 1H), 1.45-1.42 (m, 2H), 0.89-0.77 (m, 1H). ^{13}C NMR (100 MHz, DMSO-d_6) δ_C: 169.2, 167.6, 151.5, 150.1 135.2, 131.2, 130.7, 128.9, 128.7, 126.2, 118.2, 118.1, 82.7, 61.3, 56.6, 32.5, 28.5, 28.6, 27.9, 25.3, 22.0. HRMS (ESI) calcd. for $C_{22}H_{22}N_4O_3$ [M + H]$^+$: 391.1692, found: 391.1681 [M+H]$^+$.

3-Amino-1-(4-cyanophenyl)-1',3'-dimethyl-2',4',6'-trioxo-2',3',4',6,6',7,8,8a-octahydro-1*H*,1'*H*-spiro[naphthalene-2,5'-pyrimidine]-4-carbonitrile, IVn ($C_{23}H_{21}NO_3$)

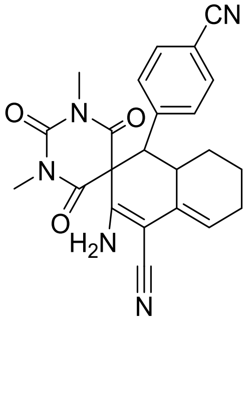

White solid; M.p.: 266-268°C (Lit.: 268-270); IR (ν_{max}, cm^{-1}) (KBr): 3452, 3336, 3308, 2234, 2211, 1679, 1639, 1603, 1458, 1381; ^1H NMR (400 MHz, DMSO-d_6) δ = 7.80-7.72 (m, 2H, ArH), 7.11-7.04 (m, 2H, ArH), 6.51 (s, 2H, NH_2), 5.58 (s, 1H, vinyl), 3.34 (m, 1H, merged with solvent), 3.16 (d, 1H, J = 12 Hz, CH), 2.87 (s, 3H, CH_3), 2.86 (s, 3H, CH_3), 2.14-2.02 (m, 2H), 1.62-1.60 (m, 1H), 1.36-1.22 (m, 2H), 0.81-0.72 (m, 1H); ^{13}C NMR (DMSO-d_6) δ = 168.26, 166.74, 150.70, 149.61, 140.83, 132.48, 132.12, 131.51, 130.39, 127.33, 118.46, 117.72, 111.19, 82.21, 60.49, 55.59, 32.02, 28.23, 28.17, 27.25, 24.89, 21.51. . HRMS (ESI) calcd. for $C_{23}H_{21}N_5O_3$ $[M + H]^+$: 416.1644, found: 416.1693 $[M+H]^+$.

3-Amino-1',3'-dimethyl-2',4',6'-trioxo-1-(thiophen-2-yl)-2',3',4',6,6',7,8,8a- octahydro-1*H*,1'*H*-spiro[naphthalene-2,5'-pyrimidine]-4-carbonitrile, IVo ($C_{20}H_{20}N_4O_3S$)

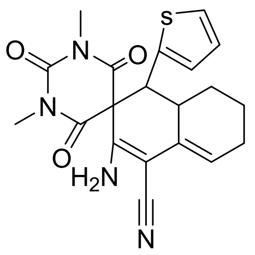

Pale green solid; M.p.: 236-238°C (Lit.: 235-237); IR (ν_{max}, cm^{-1}) (KBr): 2201, 1652, 1378; ^1H NMR (400 MHz, DMSO-d_6) δ = 7.43-7.41 (m, 1H, ArH), 6.91 (bs, 1H, ArH), 6.69-6.68 (m, 1H, ArH), 6.51 (s, 2H, NH_2), 5.56 (s, 1H, vinyl), 3.19 (s, 2H), 2.95 (s, 3H, CH_3), 2.92 (s, 3H, CH_3), 2.15-2.05 (m, 2H), 1.67-1.64 (m, 1H), 1.45-1.31 (m, 2H), 0.84-0.75 (m, 1H); ^{13}C NMR (DMSO-d_6) δ = 169, 167.1, 166.2, 150.7, 150.2, 149.9, 130.5, 130.1, 126, 124.8, 117.5, 82.0, 60.7, 59.2, 37.1, 34.2, 28.3, 27.2, 25, 21.5; HRMS (ESI) calcd. for $C_{20}H_{20}N_4O_3S$ $[M + H]^+$: 397.1256, found: 397.1334 $[M+H]^+$.

3-Amino-1',3'-dimethyl-2',4',6'-trioxo-1-(4-(trifluoromethyl)phenyl)-1',3',4',6,6',7,8,8a-octahydro-1H,2'H-spiro[naphthalene-2,5'-pyrimidine]-4-carbonitrile, IVp ($C_{23}H_{21}F_3N_4O_3$)

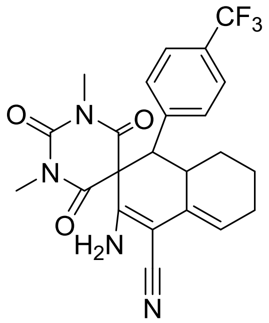

White solid; M.p.: 248-250°C; IR (v_{max}, cm^{-1}) (KBr): 2203, 1675, 1432, 1378, 752; ^1H NMR (400 MHz, DMSO-d_6) δ = 7.78 (d, 1H, *J*= 8.0 Hz, Ar-H), 7.71 (d, 1H, *J*=8.0 Hz, Ar-H), 7.24 (d, 1H, *J*= 8.0 Hz, Ar-H), 7.18 (d, 1H, *J*=8.0 Hz, Ar-H), 6.49 (s, 2H, -NH_2), 5.69 (s, 1H, vinylic), 3.25-3.23 (m, 2H, -CH), 2.96-2.94 (m, 6H, 2(CH_3)), 2.25-2.04 (m, 2H), 1.74-1.71 (m, 1H), 1.46-1.44 (m, 1H), 1.13-1.1 (m, 1H), 0.89-0.83 (m, 1H); ^{13}C NMR (DMSO-d_6) δ = 169.9, 167.2, 151.2, 150, 131.7, 130.8, 127.5, 125.3, 118.2, 117.9, 82.2, 61, 56.5, 56, 32.5, 28.6, 27.8, 25.3, 21.9, 27.2, 18.9; HRMS (ESI) calcd. for $C_{23}H_{21}F_3N_4O_3$: 459.1567 [M+H]; found: 459.1620 [M+H]+

Scanned spectra of representative compounds

3-Amino-1',3'-dimethyl-2',4',6'-trioxo-1-*p*-tolyl-2',3',4',6,6',7,8,8a-octahydro-1*H*,1'*H*spiro[naphthalene-2,5'-pyrimidine]-4-carbonitrile, IVb ($C_{23}H_{24}N_4O_3$)

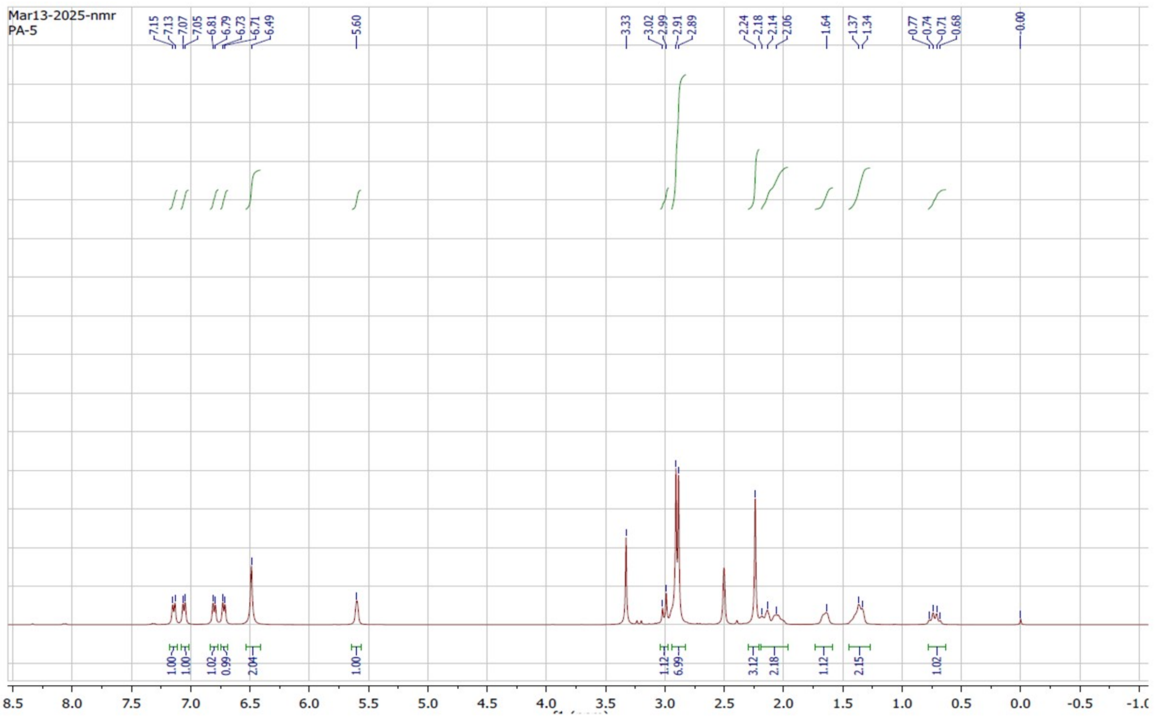

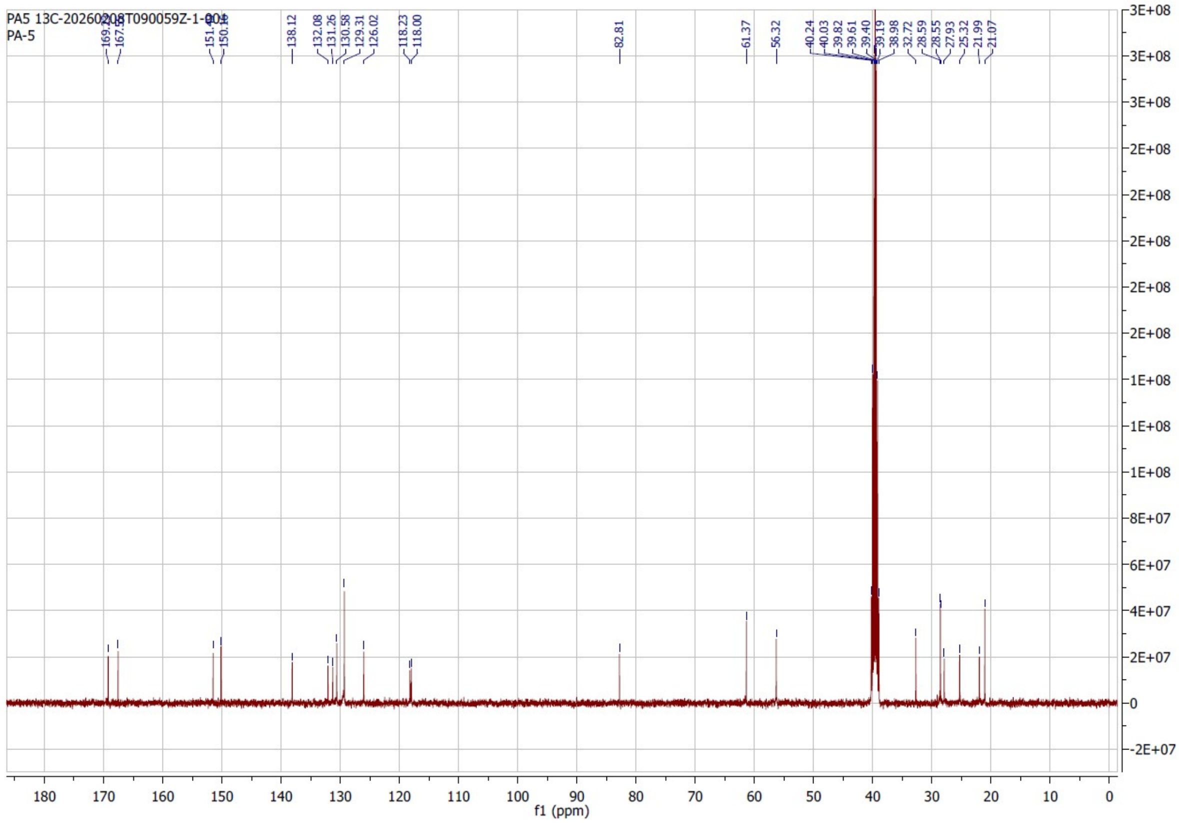

3-Amino-1-(4-chlorophenyl)-1',3'-dimethyl-2',4',6'-trioxo-1',3',4',6,6',7,8,8a-octahydro-1H,2'H-spiro[naphthalene-2,5'-pyrimidine]-4-carbonitrile, IVc ($C_{22}H_{21}ClN_4O_3$)

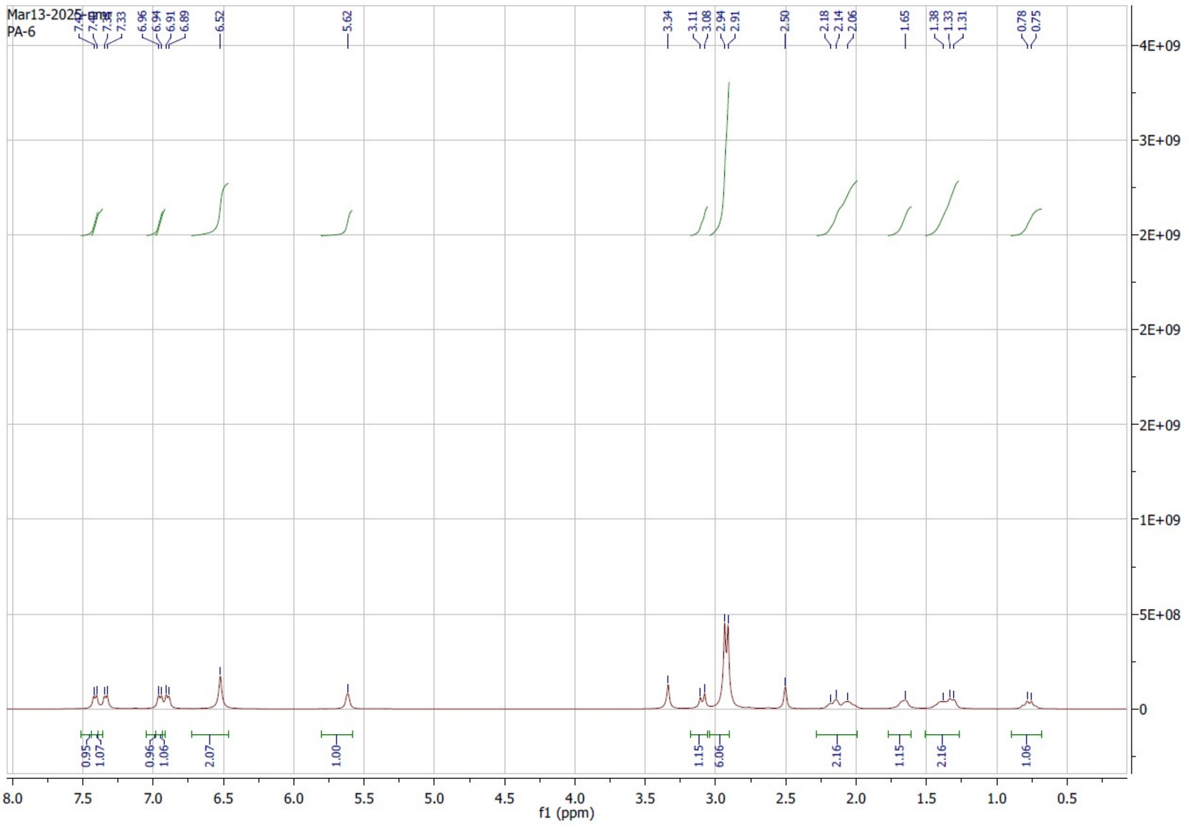

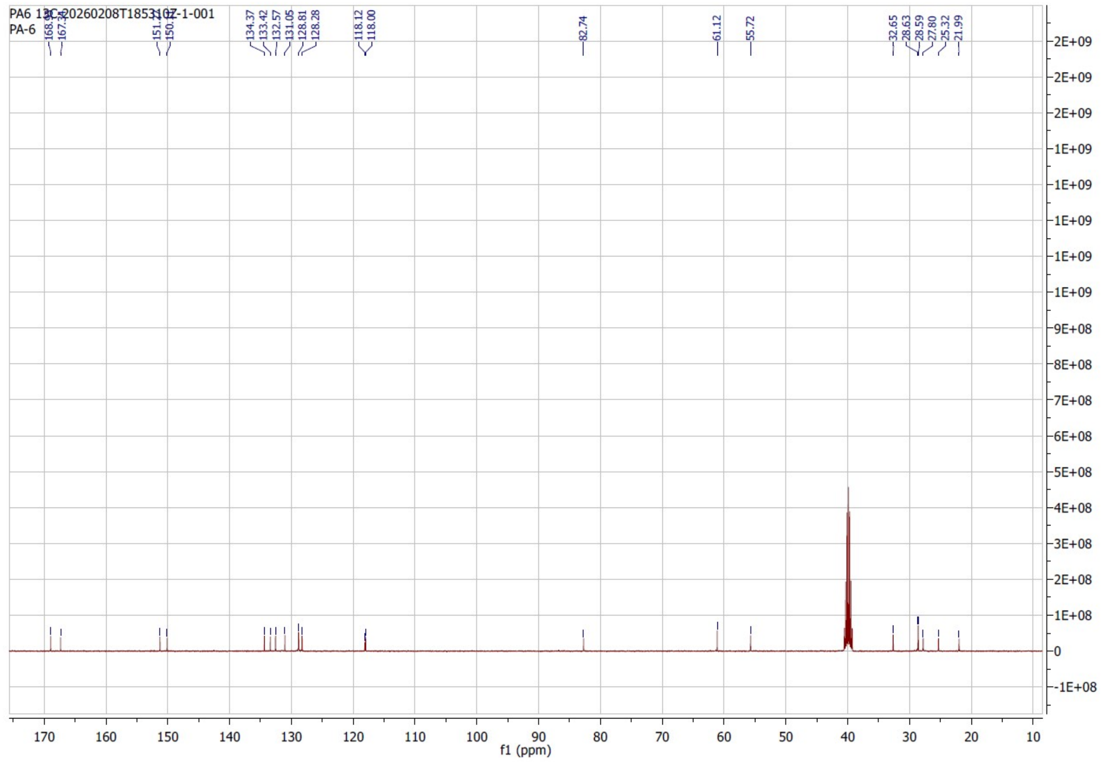

3-Amino-1-(4-bromophenyl)-1',3'-dimethyl-2',4',6'-trioxo-1',3',4',6,6',7,8,8a-octahydro-1*H*,2'*H*-spiro[naphthalene-2,5'-pyrimidine]-4-carbonitrile, IVf ($C_{22}H_{21}BrN_4O_3$)

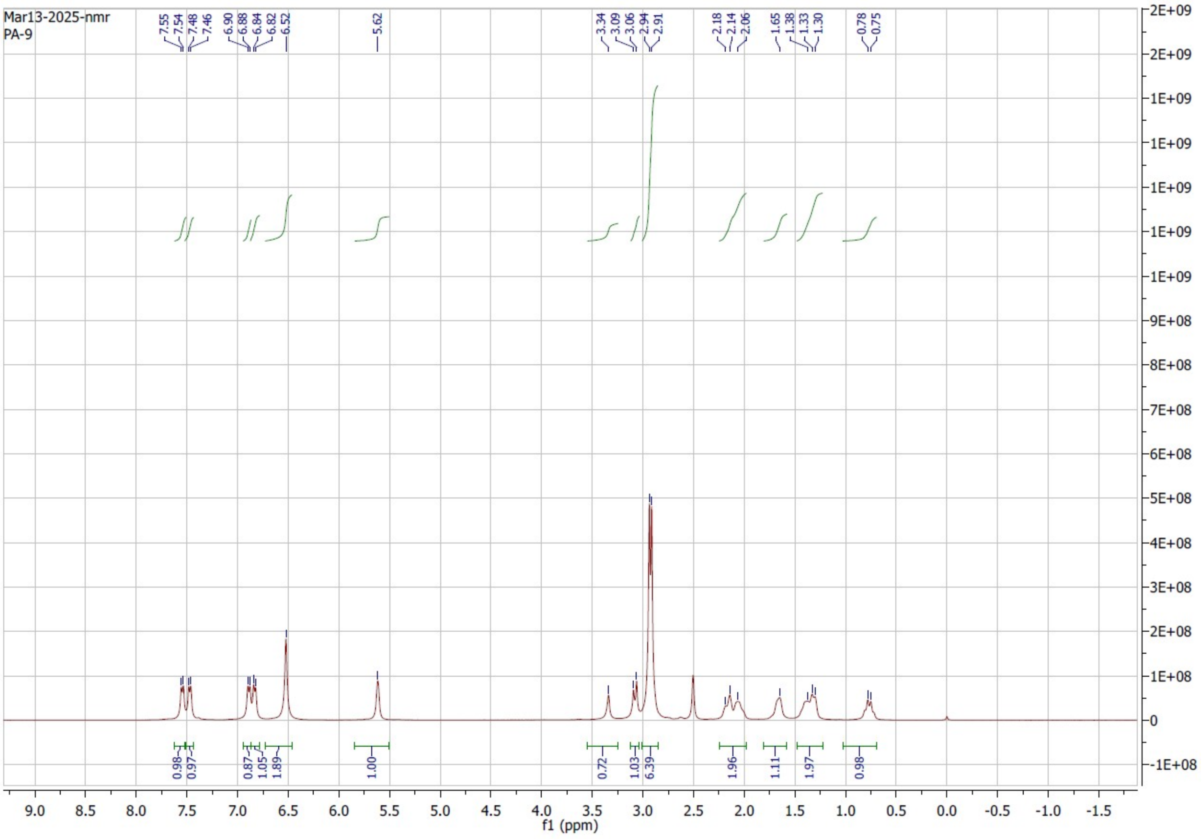
Mar13-2025-nmr
PA-9
7.55
7.54
7.48
7.46
6.90
6.88
6.84
6.82
6.52
5.62
3.34
3.09
3.06
2.94
2.91
2.18
2.14
2.06
1.65
1.38
1.33
1.30
0.78
0.75
0.98
0.97
0.87
1.05
1.89
1.00
0.72
1.03
6.39
1.96
1.11
1.97
0.98
f1 (ppm)

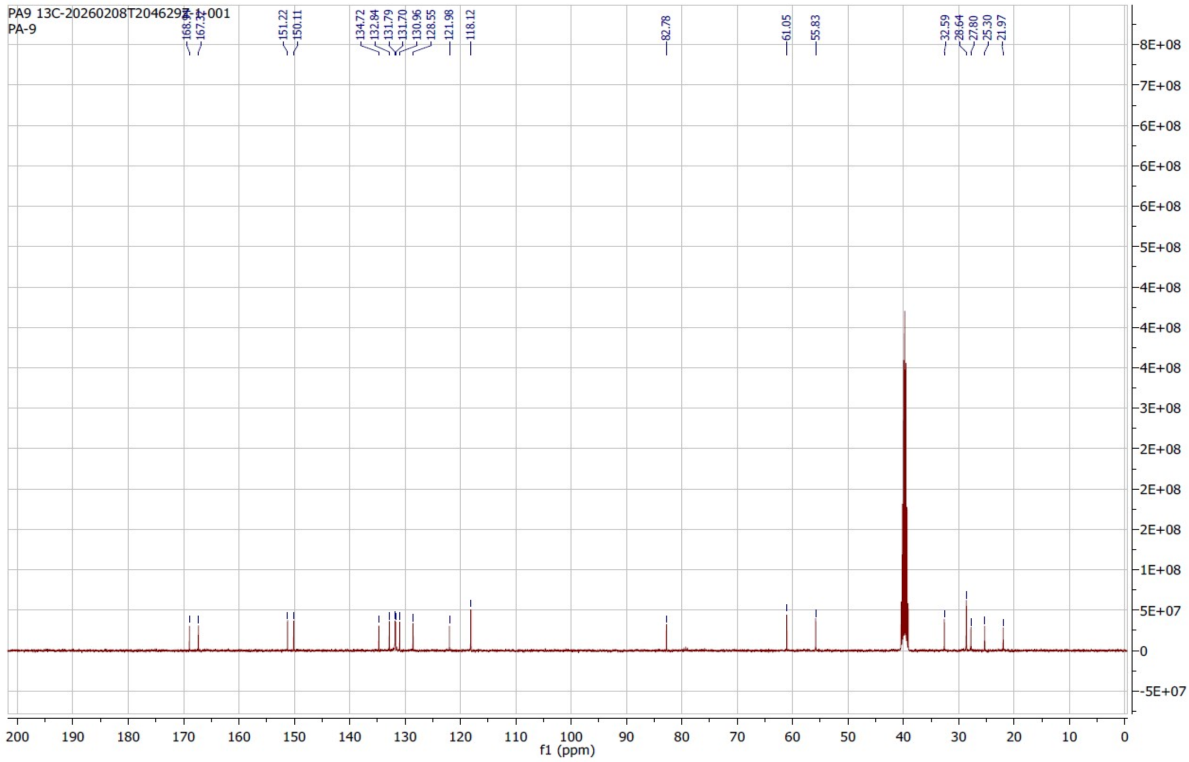
PA9 13C-20260208T204629Z-1-001
PA-9
168.94
167.32
151.22
150.11
134.72
132.84
131.79
131.70
130.96
128.55
121.98
118.12
82.78
61.05
55.83
32.59
28.64
27.80
25.30
21.97
f1 (ppm)

3-Amino-1',3'-dimethyl-2',4',6'-trioxo-1-(3-bromophenyl)-2',3',4',6,6',7,8,8a-octahydro-1*H*,1'*H*spiro[naphthalene-2,5'-pyrimidine]-4-carbonitrile, IVh ($C_{22}H_{21}BrN_4O_3$)

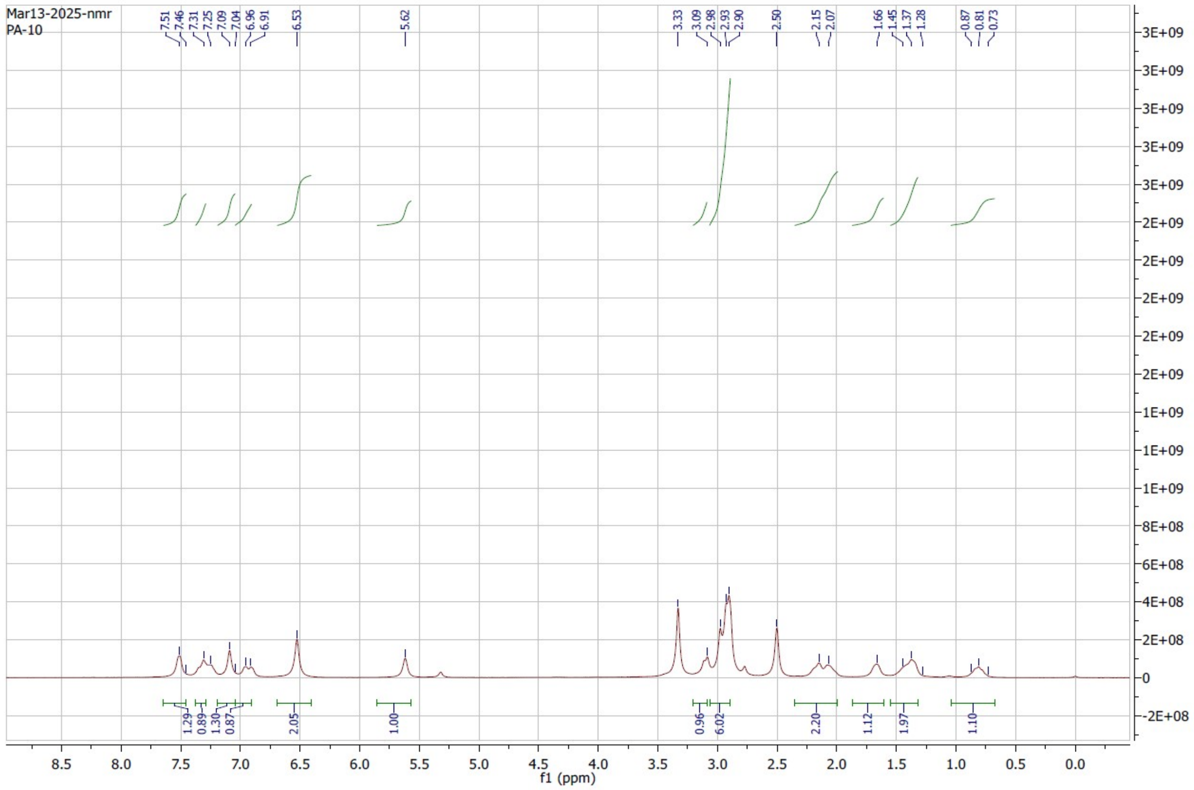

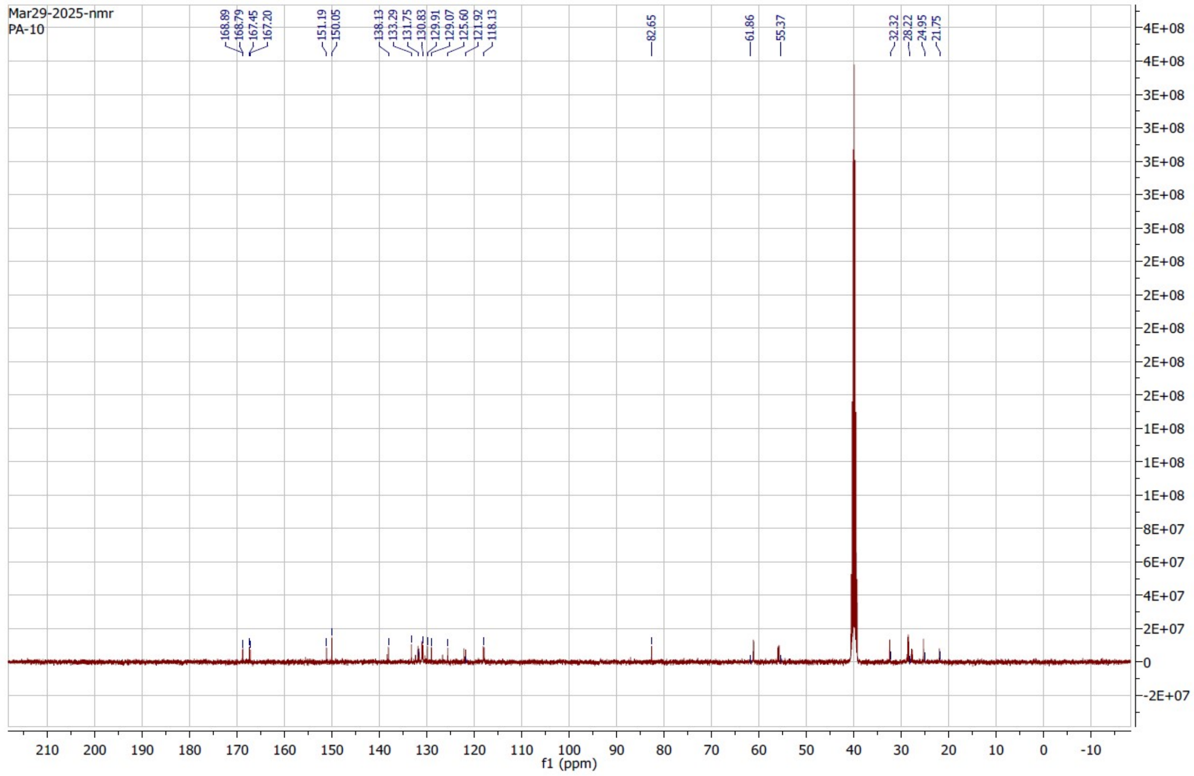

3-Amino-1-(2-chlorophenyl)-1',3'-dimethyl-2',4',6'-trioxo-2',3',4',6,6',7,8,8a-octahydro-1H,1'H-spiro[naphthalene-2,5'-pyrimidine]-4-carbonitrile, IVo ($C_{22}H_{21}N_4O_3$)

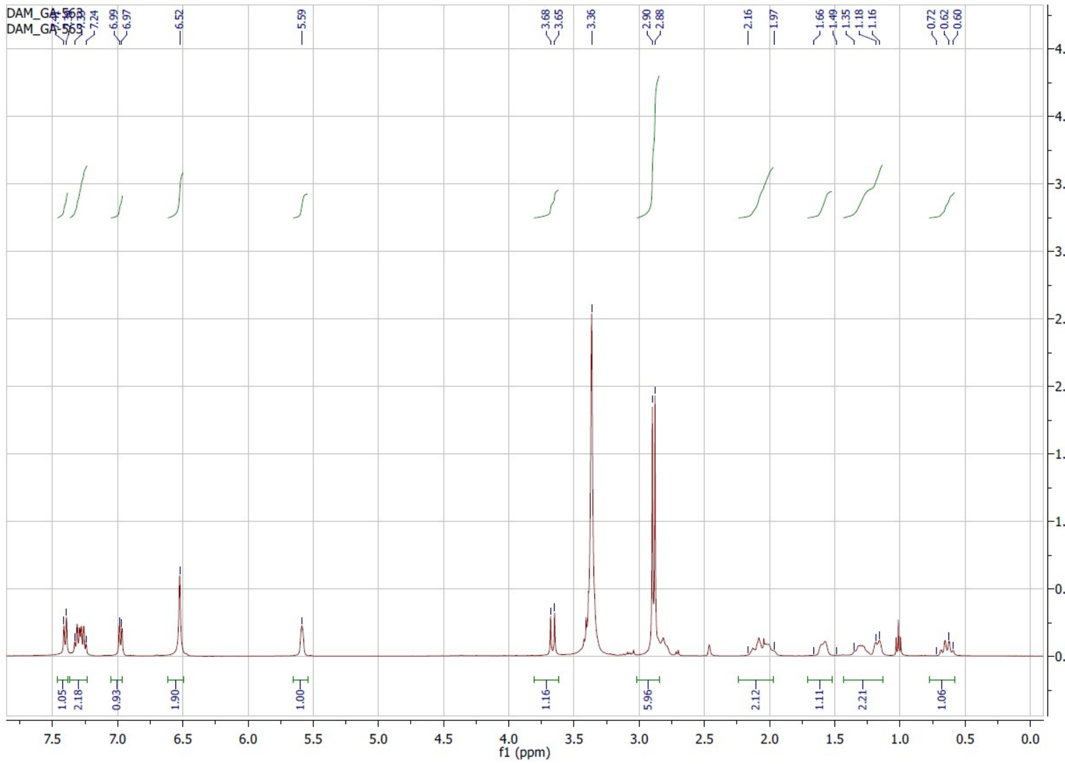

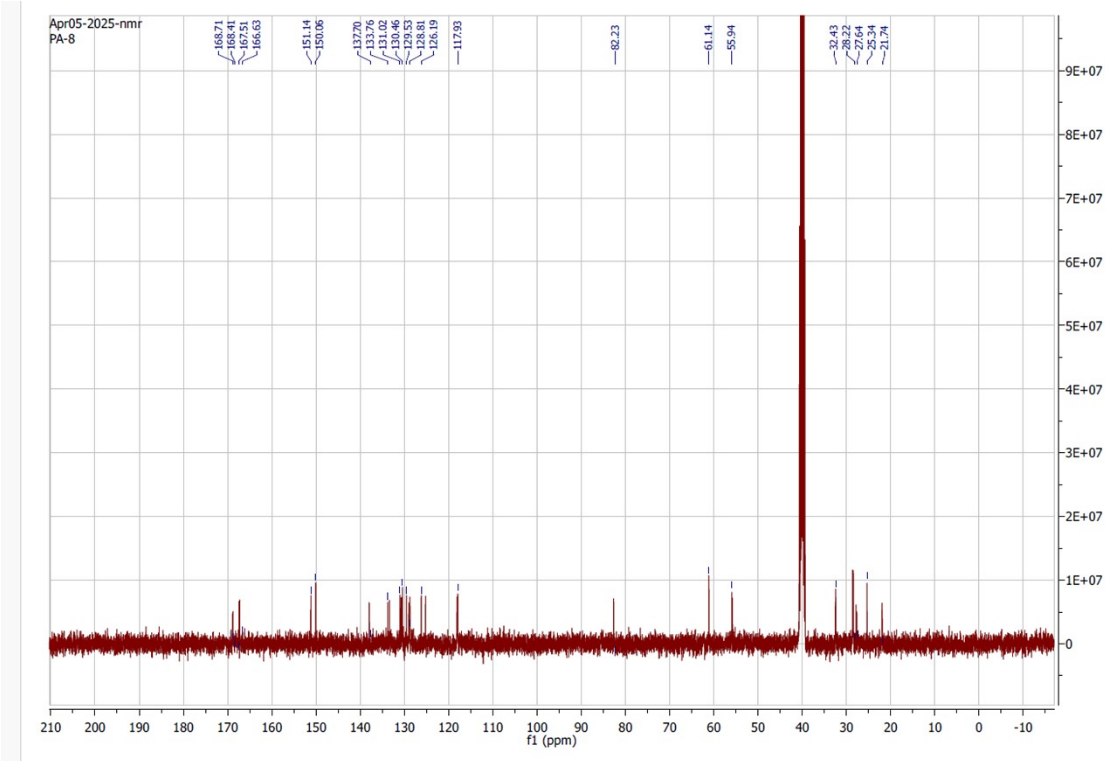

3-Amino-1',3'-dimethyl-2',4',6'-trioxo-1-(3,4,5-trimethoxyphenyl)-2',3',4',6,6',7,8,8a-octahydro-1*H*,*1'H*-spiro[naphthalene-2,5'-pyrimidine]-4-carbonitrile, IVj

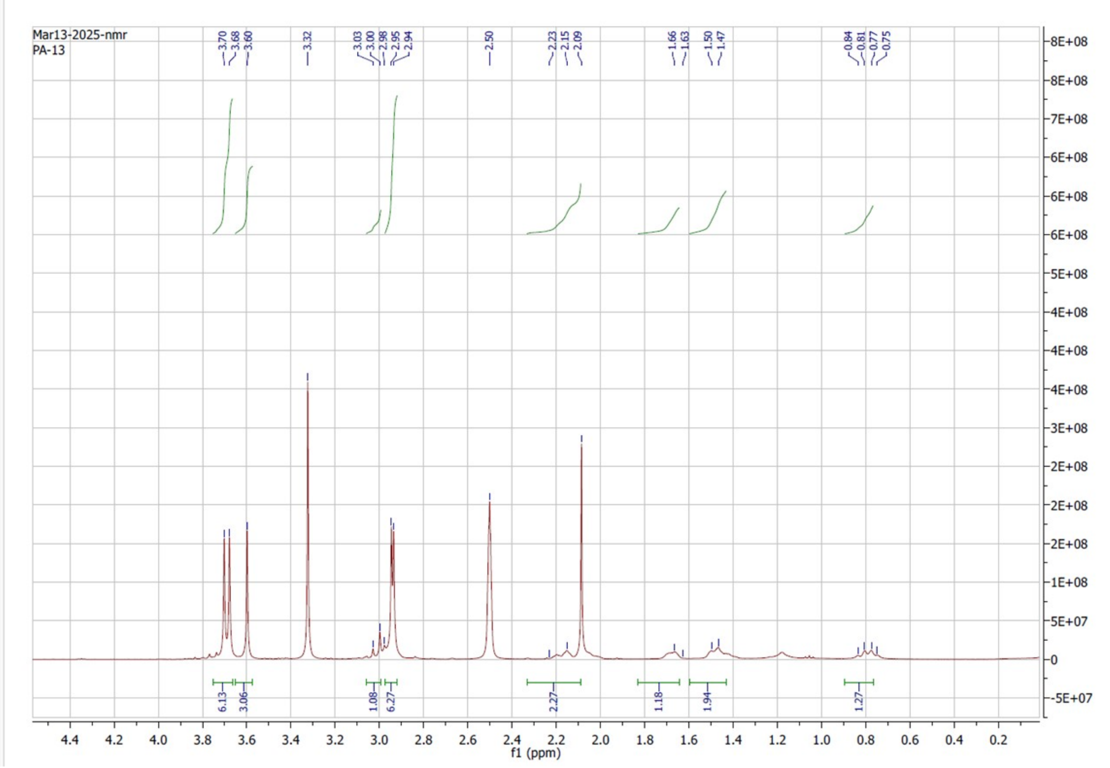

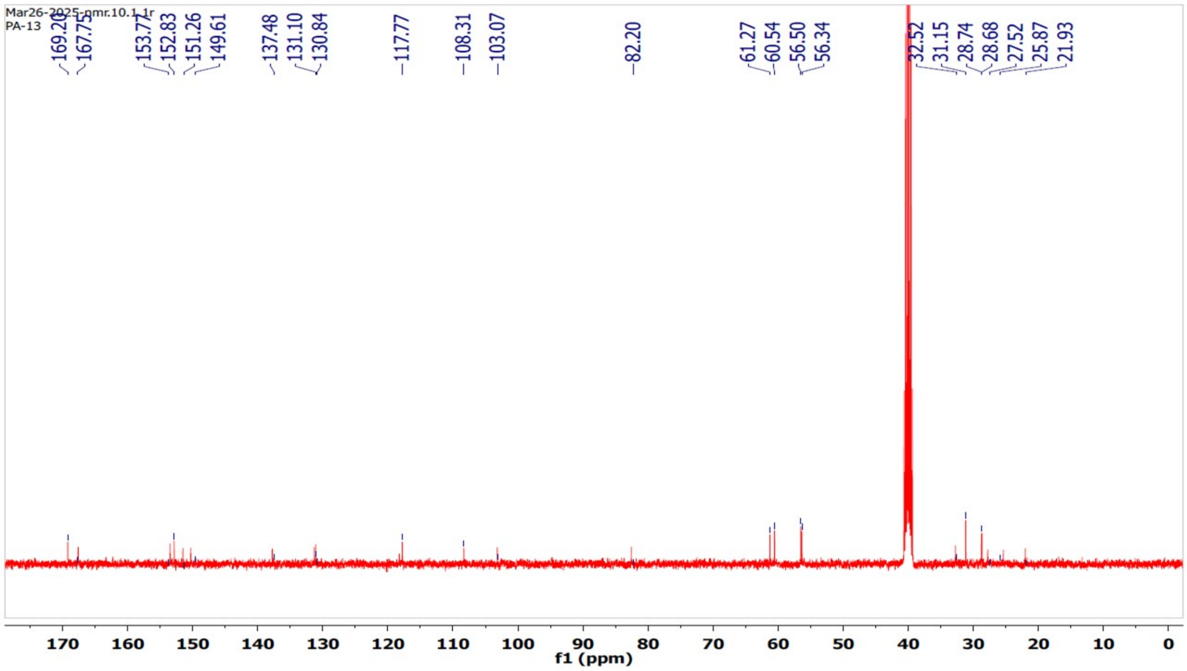

3-amino-1-(3-methoxyphenyl)-1',3'-dimethyl-2',4',6'-trioxo-2',3',4',6,6',7,8,8a-octahydro1*H*,1'*H*-spiro[naphthalene-2,5'-pyrimidine]-4-carbonitrile, IVk

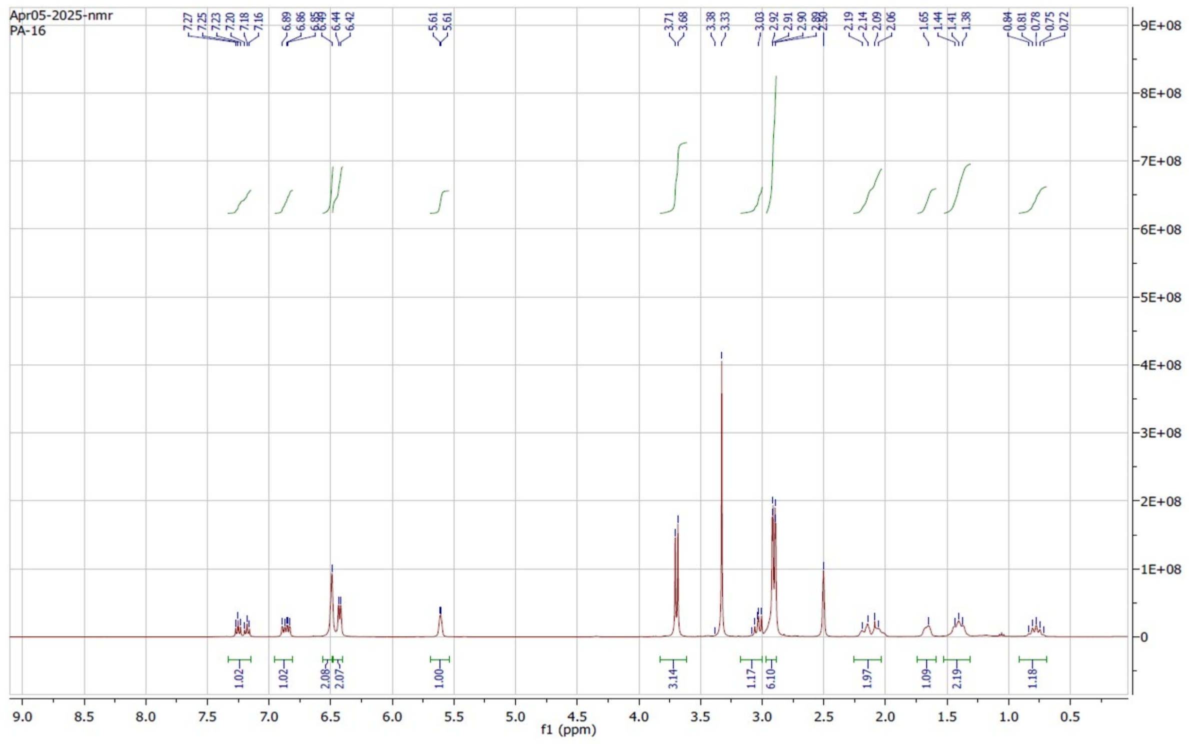

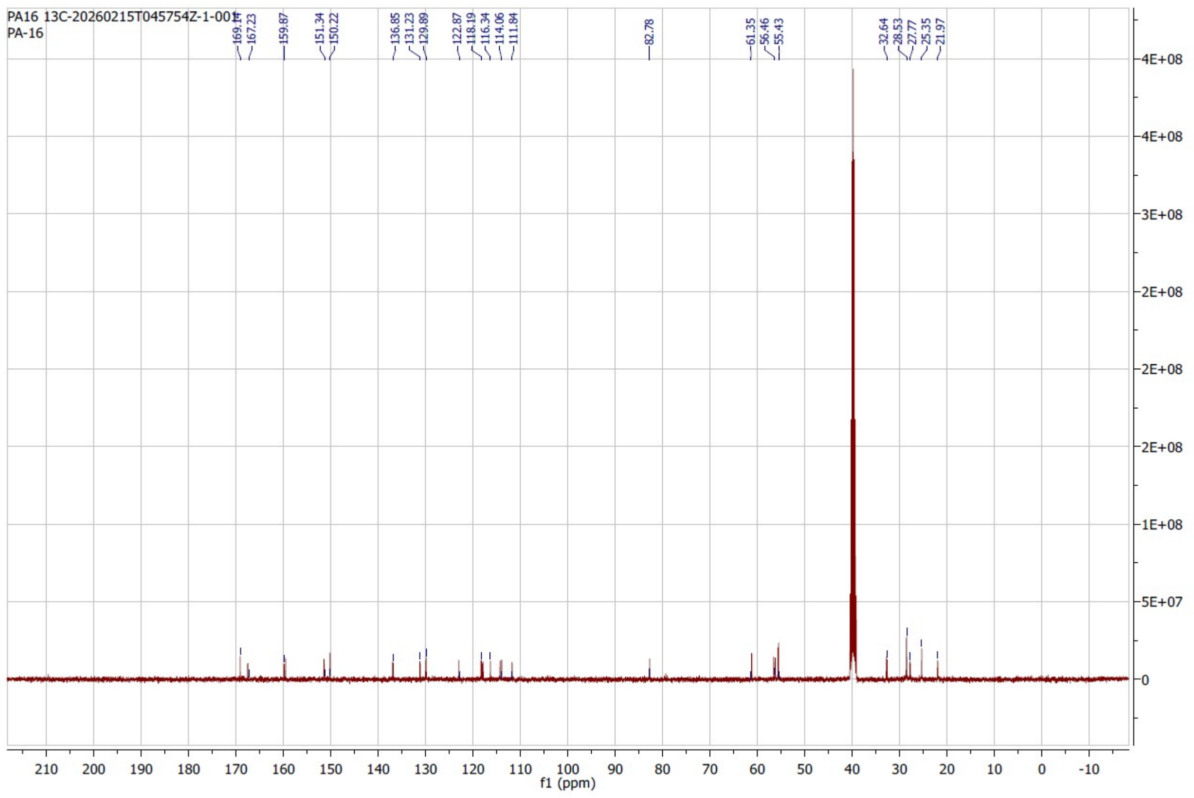

Supplement: RA-016-D6RA02921C-s001 [file RA-016-D6RA02921C-s001.pdf]
